# Mitochondrial changes associated with viral infectious diseases in the paediatric population

**DOI:** 10.1002/rmv.2232

**Published:** 2021-03-31

**Authors:** Sonia Romero‐Cordero, Antoni Noguera‐Julian, Francesc Cardellach, Clàudia Fortuny, Constanza Morén

**Affiliations:** ^1^ Faculty of Medicine Pompeu Fabra University Barcelona Spain; ^2^ Faculty of Medicine Universitat Autònoma de Barcelona Bellaterra Spain; ^3^ Malalties Infeccioses i Resposta Inflamatòria Sistèmica en Pediatria Unitat d´Infeccions Servei de Pediatria Institut de Recerca Pediàtrica Hospital Sant Joan de Déu Barcelona Spain; ^4^ Departament de Pediatria Universitat de Barcelona Barcelona Spain; ^5^ CIBER de Epidemiología y Salud Pública, CIBERESP (ISCIII) Madrid Spain; ^6^ Red de Investigación Translacional en Infectología Pediátrica RITIP Madrid Spain; ^7^ Faculty of Medicine and Health Sciences Muscle Research and Mitochondrial Function Laboratory Cellex‐IDIBAPS University of Barcelona Barcelona Spain; ^8^ CIBER de Enfermedades Raras CIBERER (ISCIII) Madrid Spain; ^9^ Internal Medicine Department Hospital Clínic of Barcelona (HCB) Barcelona Spain

**Keywords:** antivirals, infections, mitochondria, paediatrics, virus

## Abstract

Infectious diseases occur worldwide with great frequency in both adults and children, causing 350,000 deaths in 2017, according to the latest World Health Organization reports. Both infections and their treatments trigger mitochondrial interactions at multiple levels: (i) incorporation of damaged or mutated proteins into the complexes of the electron transport chain; (ii) impact on mitochondrial genome (depletion, deletions and point mutations) and mitochondrial dynamics (fusion and fission); (iii) membrane potential impairment; (iv) apoptotic regulation; and (v) generation of reactive oxygen species, among others. Such alterations may result in serious adverse clinical events with considerable impact on the quality of life of the children and could even cause death. Herein, we use a systematic review to explore the association between mitochondrial alterations in paediatric infections including human immunodeficiency virus, cytomegalovirus, herpes viruses, various forms of hepatitis, adenovirus, T‐cell lymphotropic virus and influenza. We analyse how these paediatric viral infectious processes may cause mitochondrial deterioration in this especially vulnerable population, with consideration for the principal aspects of research and diagnosis leading to improved disease understanding, management and surveillance.

**Abbreviations**
3TC: Lamivudine
ABC: Abacavir
ADF: Adefovir
ADP: Adenosine diphosphate
AIDS: Acquired immunodeficiency syndrome
ALT: Alanine aminotransferase
ANT: Adenine nucleotide translocator
AST: Aspartate aminotransferase
ARV: Antiretrovirals
ATLL: Adult T‐cell leukaemia/lymphoma
ATP: Adenosine triphosphate
ATV: Atazanavir
CI: Complex I
CII: Complex II
CIII: Complex III
CIV: Complex IV
CV: Complex V
Ca^2+^
: Calcium
CD: Cluster of differentiation
CNS: Central nervous system
COBI: Cobicistat
CoQ: Coenzyme Q
CytC: Cytochrome C
D4T: Stavudine
DdC: Zalzitabine
DdI: Didanosine
DNA: Deoxyribonucleic acid
Drp1: Dynamin related protein 1
DOR: Doravirine
DRV: Darunavir
DTG: Dolutegravir
EFV: Efavirenz
EI: Entrance inhibitors
ER: Endoplasmic reticulum
ETC: Electron transport chain
ETR: Etravirine
EVG: Elvitegravir
FADH: Flavine and adenine dinucleotide hydrogen
FDA: Food and drug administration
FI: Fusion inhibitors
FTC: Emtricitabine
Gp: Glycoprotein
HAV: Hepatitis A virus
HBV: Hepatitis B virus
HCV: Hepatitis C virus
HDV: Hepatitis D virus
HHV‐8: Human herpesvirus type 8
HSV: Herpes simplex virus
IBA: Ibalizumab‐uiyk
IFN: Interferon
H_2_O: Water
H_2_O_2_
: Hydrogen peroxide
HCMV: Human cytomegalovirus
HIV: Human immunodeficiency virus
HTLV‐1: Human T‐cell lymphotrophic virus
II: Integrase inhibitors
IMM: inner mitochondrial membrane
IP3: Inositol triphosphate
KSHV: Kaposi sarcoma‐associated herpesvirus
LPV: Lopinavir
MAM: Mitochondrial associated membrane
MAVS: Mitochondrial antiviral signalling protein
Mfn: Mitofusin
MRC: Mitochondrial respiratory chain
MtDNA: Mitochondrial DNA
MVC: Maraviroc
NADH: Nicotinamide adenine dinucleotide
NF‐KB: Nuclear factor kappa enhancer of B‐cell light chain activated KB
NMDA: N‐methyl‐D‐aspartic acid
NO: Nitric oxide
NOS: Nitric oxide synthase
NRTI: Nucleoside/nucleotide reverse transcriptase inhibitors
NNRTI: Non‐nucleoside reverse transcriptase inhibitors
NVP: Nevirapine
O_2_^—,^
Superoxide anion
OH: Hydroxyl anion
OMM: Outer mitochondrial membrane
ONOO^−^
: Peroxynitrite
OXPHOS: Oxidative phosphorylation system
PCR: Polymerase chain reaction
PI: Protease inhibitors
PTP: Permeability transition pore
PUL37x1: UL37 exon 1 protein
RAL: Raltegravir
RNA: Ribonucleic acid
R‐OOH: Hydroperoxides
ROS: Reactive oxygen species
RPV: Rilpivirine
RTV: Ritonavir
SOD: Superoxide dismutase
SQV: Saquinavir
T20: Enfuvirtide
TCA: Tricarboxylic acid
TDF: Tenofovir
TLR: Toll‐like receptor
TNAV: HIV‐associated neurocognitive disorder
TPV: Tripanavir
VDAC: Voltage‐dependent anion channel
VMIA: Apoptosis viral inhibitor in mitochondria
WHO: World Health Organization
ZDV: Zidovudine

## 1 INTRODUCTION

Our multidisciplinary team is composed of basic and translational researchers (mitochondriologists) as well as clinicians (experts in infectious diseases, paediatrics and internal medicine). Our expertise is therefore focused precisely on the three main fields carefully covered in this review: (i) mitochondrial metabolism, (ii) infectious diseases and (iii) paediatrics.

The fact that the activities of our team are mainly focused on the above‐mentioned issues, has allowed us to coordinate on this common project over several months. There is an urgent need to depict mitochondrial alterations derived from infective processes in the paediatric population, as mitochondrial status has a great impact on the severity and progression of disease. As far as we are aware, this is the first time that mitochondrial impairment related to both viral infections and the anti‐viral agents used for treatment in paediatrics has been reviewed. Also, special focus has been placed on children, as the most vulnerable population group.

### 1.1 Mitochondria

Mitochondria are semi‐autonomous, maternally inherited organelles present in the cytoplasm of virtually all eukaryotic cells.^1^, ^2^ They are essential for cell viability, due to their involvement in cellular respiration, apoptosis, catabolism and anabolism of metabolites, calcium homeostasis, thermogenesis, and, through the formation of adenosine triphosphate (ATP) molecules, energy production.^3^ They are present in a variable number within cells depending on the energy requirements of each specific tissue. The greater the energy demand is, the greater the number of mitochondria, with the greatest numbers present in nervous and muscular tissues.^4^

**FIGURE 1:**
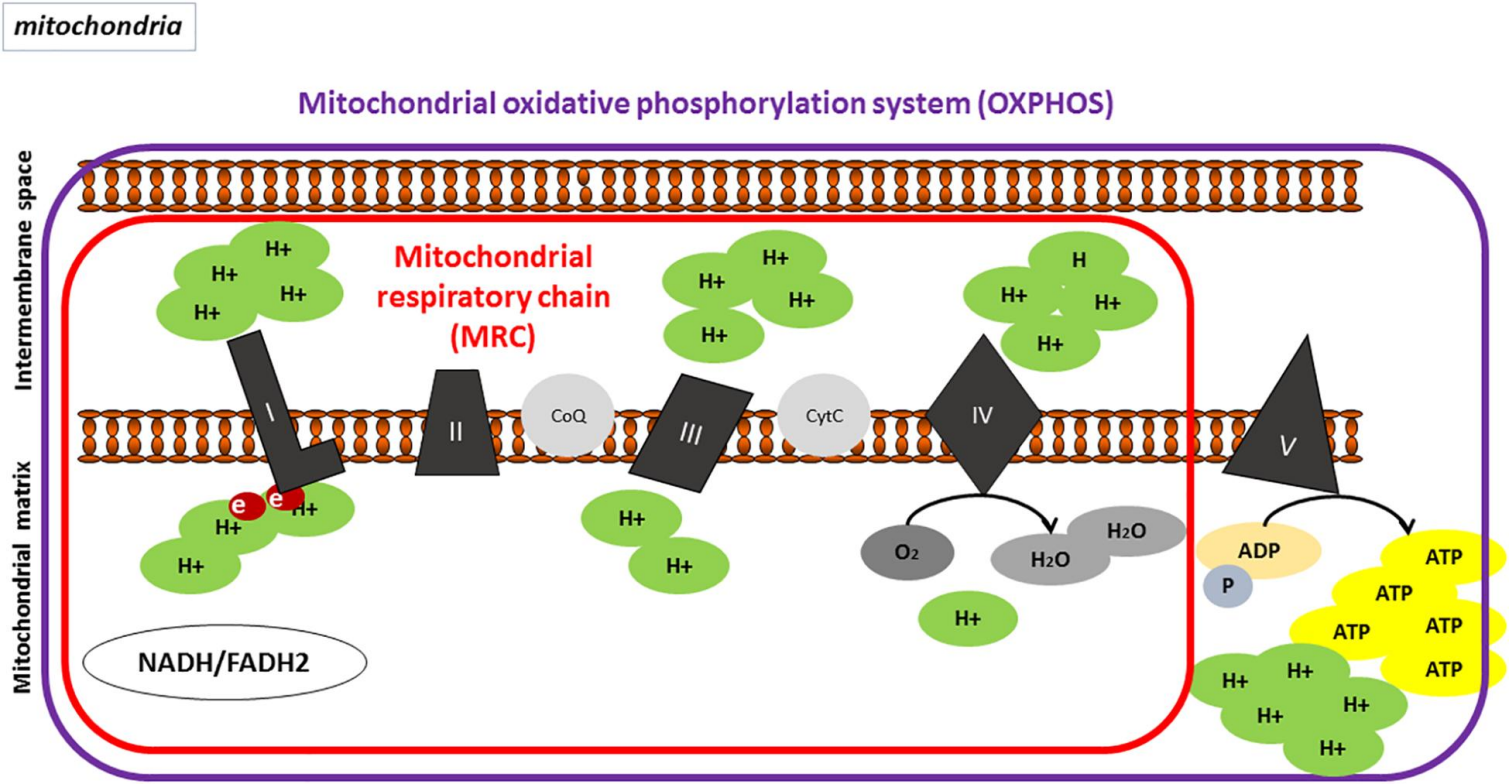
Mitochondrial respiratory chain and oxidative phosphorylation system, located in the inner mitochondrial membrane. Oxidative phosphorylation is the synthesis process of ATP coupled to oxygen consumption, through the transfer of electrons in stages. The electrons flow through the MRC through oxidation–reduction (or redox) reactions ending in complex IV, where oxygen is the final receptor for the electrons and is reduced to H_2_O. In the OXPHOS, oxygen is consumed and an electrochemical gradient is established, driving ATP synthesis. ADP, adenosine diphosphate; ATP, adenosine triphosphate; CoQ, co‐enzyme Q; CytC, cytochrome C; e, electrons; FADH, flavin and adenine dinucleotide; H+, proton; I, I complex; II, II complex; III, III complex; IV, IV complex; MRC, mitochondrial respiratory chain; NADH, Nicotinamide adenine dinucleotide hydrogen; OXPHOS, oxidative phosphorylation system; V, V complex

Mitochondria are not static structures within cells, but are dynamic, capable of merging and fissioning. They consist of (i) the outer mitochondrial membrane (OMM), permeable to ions, metabolites and polypeptides, due to porins and/or voltage‐dependent channels^2^; (ii) the inner mitochondrial membrane (IMM), impervious to almost all molecules and ions, highly selective and rich in cardiolipin and consisting of folds, shaping the mitochondrial cristae, where the enzymatic complexes of the oxidative phosphorylation system (OXPHOS) are embedded^4^ (Figure 1); (iii) the intermembrane space between OMM and IMM; (iv) the mitochondrial matrix containing ions, oxidizable metabolites and the genetic material of the mitochondria, and the mitochondrial DNA (mtDNA).

#### 1.1.1 Mitochondrial physiology

Mitochondria respond to a series of genetic, metabolic and neuroendocrine signals through functional and morphological changes, and in turn generate signals that influence a large number of cellular functions that contribute to the complexity of physiology and pathology. This places the mitochondria in a privileged position, as a “portal” at the intersection of the cell and its environment.^5^ Thus, mitochondria have been implicated in ageing, regulation of cell metabolism, control of the cell cycle, cell development, antiviral responses, signal transduction, among others.^6^ The tricarboxylic acid (TCA) cycle, also called the Krebs cycle or the citric acid cycle, which takes place within the matrix of the mitochondria, is a series of eight enzymatic steps that consume, and then regenerate, citrate. It links the metabolism of carbohydrates, fats and proteins, since the catabolism of these compounds generates acetyl‐CoA. This key molecule enters the TCA cycle, oxidizes, producing flavin and adenine dinucleotide hydrogen (FADH) and nicotinamide adenine dinucleotide (NADH), reducing molecules that will feed the mitochondrial respiratory chain (MRC) and OXPHOS.^7^

#### 1.1.2 Mitochondrial pathology

The first patient with mitochondrial disease was described in 1962.^8^ Human mitochondrial diseases are actually a very large collection of hundreds of very heterogeneous and rare diseases, since changes in any of literally thousands of genes can affect the mitochondrial function.^7^ Hence, mitochondrial research is on the rise in the medical sciences. As evidence, the number of medical publications related to mitochondriopathies has surpassed those related to other alterations in other organelles, including the endoplasmic reticulum (ER), the Golgi apparatus and the nucleus.^5^ Mitochondrial disorders represent a major challenge in medicine.^8^ Similarly, the origin of pleiotropic and multisystemic symptoms in mitochondrial disorders is still poorly understood and often makes it difficult to diagnose this group of diseases.^5^ Oxidative tissues, with high energy demand (including the brain, muscle, retina, cochlea, liver and kidney) are the most vulnerable to OXPHOS defects.^8^ Clinical presentations in childhood include allergy, hypotonia, development of mental retardation, conduction failure, seizures, cardiomyopathy, hearing or visual impairment, movement disorders and lactic acidosis.^9^

##### Anaerobiosis

In abnormal conditions, such as hypoxia or alterations in mitochondrial function, metabolic pathways are readjusted to continue obtaining the reducing power responsible for energy production through anaerobic processes. Under these conditions, the pyruvate resulting from the catabolism of metabolites is not imported into the mitochondria, but is instead converted to lactate by the enzyme lactate dehydrogenase. In this pathological context, lactate concentration increases in the bloodstream, originating from its synthesis in the skeletal muscle, liver, nervous and lymphoid tissues. Under normal conditions, serum lactate concentration ranges from 0.5 to 2.4 mmol/L.^4^ In conditions of increased lactate levels, the blood pH falls and acidification occurs.^10^ A shift to anaerobic metabolism and mitochondrial dysfunction has been reported in adipose tissue of human immunodeficiency virus (HIV)‐infected patients receiving antiretrovirals (ARV).^11^

##### Reactive oxygen species

Reactive oxygen species (ROS) are intermediate metabolites derived from oxygen, and most are generated in the mitochondria during OXPHOS dysfunction. These species are free radicals (some of their electrons are decoupled) and are considered highly oxidizing, unstable and capable of damaging most cellular molecules and structures, such as proteins, lipids, carbohydrates, genetic material and mitochondria, which are particularly vulnerable. Some examples of ROS are superoxide anion (O_2_^−^) and hydrogen peroxide (H_2_O_2_), which are relatively stable, although hydroxyl anion (OH^−^) and peroxynitrite (ONOO^−^) are highly reactive. All of them are derived from O_2_^−^, which is mainly generated in MRC complexes I (CI) and III (CIII) through redox reactions of coenzyme Q (CoQ), which in its semi‐reduced form is capable of auto‐oxidizing and returning to its oxidized form, transferring an electron and converting molecular oxygen to O_2_^−^. There are many antioxidants, such as superoxide dismutase (SOD), capable of converting O_2_^−^ to H_2_O_2_; catalase or peroxidase can convert H_2_O_2_ to H_2_O; and glutathione peroxidase, which catalyses the conversion of H_2_O_2_, hydroperoxides (R‐OOH) and peroxide lipids to H_2_O. There are also many non‐enzymatic antioxidants, molecules such as vitamins E and C, carotenes, quinones, glutathione and metallic elements, such as selenium, zinc, iron, or copper, among others, which are capable of reducing the ROS levels. Under physiological conditions, all antioxidant mechanisms minimize ROS production and therefore act as protective systems against oxidative stress. However, in the presence of mitochondrial dysfunction, ROS increase beyond the detox threshold. ROS release may be associated with the presence of exogenous toxic compounds affecting the mitochondria. For example, an increase in ROS production linked to viral agents, such as HIV infection and ARV exposure, has been described.^4^, ^10^, ^12^, ^13^

##### Apoptosis

Apoptosis is programmed cell death, a mechanism capable of eliminating unwanted cells in three main circumstances: (a) development and homeostasis, (b) as a defence mechanism against genetic damage and potential tumour cells, and (c) natural senescence and ageing. Mitochondria play a central role in this process, characterized by cell contraction, chromatin degradation and by providing energy to the nucleus. Apoptosis processes are usually mediated by serine proteases called caspases, which are synthesized as zymogens (procaspases). Depending on the site of action, apoptotic caspases can be classified as initiators or effectors and three classic signalling pathways that lead to apoptosis are recognized: (i) the receptor or extrinsic pathway in which the stimulus is external and received by a cell surface receptor, (ii) the mitochondrial or intrinsic pathway in which the stimulus is internal and is regulated by the mitochondria,^14^ and (iii) stress mediated in the ER.

Importantly, mitochondrial alterations can be classified as primary or genetic, when the origin is a genetic alteration, which affects a mitochondrial protein; or secondary or acquired, when the cause is external or environmental, due to the presence of a toxic agent, for example, cholesterol‐lowering statin drugs,^1^ or the presence of an infectious agent, such as HIV or human cytomegalovirus (HCMV), among others.

In this article, we will review those viral infections that involve the mitochondria, per se or by their treatment toxicity, and that can be considered of relevance in the paediatric population, taking into account the principal aspects of research and diagnosis leading to improved disease understanding, management and surveillance.

## 2 VIRAL INFECTIONS AND MITOCHONDRIAL INVOLVEMENT

### 2.1 Human immunodeficiency virus

HIV belongs to the genus *Lentivirus* in the family *Retroviridae*.^15^ Two types of genetically and antigenically different viruses are known as HIV‐1 and HIV‐2. The vast majority of HIV infections in the global pandemic are caused by HIV‐1. Most HIV‐2 cases are confined to some West African countries with their epicentre in Guinea‐Bissau.^16^

HIV is present in body fluids as free virus particles and within infected immune cells and causes acquired immunodeficiency syndrome (AIDS). It primarily infects CD4^+^ T cells, macrophages and dendritic cells, in order to carry out its replication cycle. HIV infection is associated with a progressive decrease in CD4^+^ T‐cell count and an increase in viral load. In the haematopoietic system, CD4^+^ T lymphocytes are the most visibly infected cell type since they express the CD4 molecule used by HIV as a receptor and can efficiently replicate the virus. Macrophages are also frequently found to be infected with HIV, but this infection may go unnoticed due to low viral production.^17^

**FIGURE 2:**
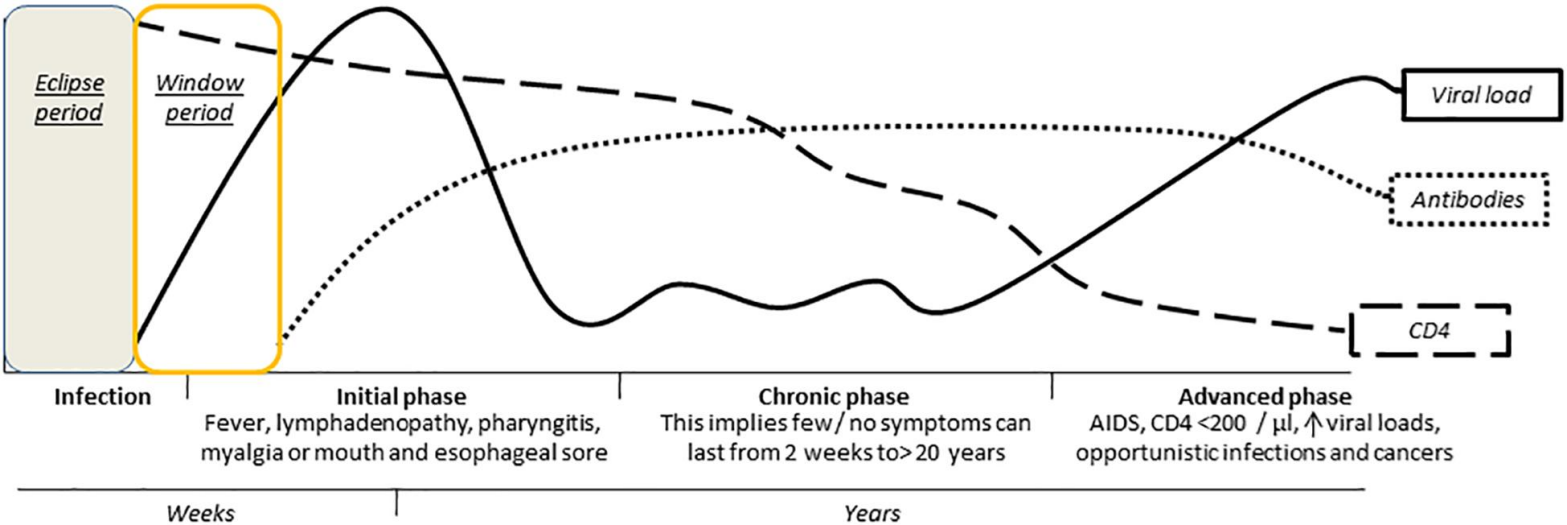
Different stages of HIV infection over time. The stages are (a) acute infection (also known as primary infection), which lasts for several weeks and it can include symptoms like fever, lymphadenopathy, pharyngitis, myalgia, or mouth and esophageal sores. (b) The latency stage involves few or no symptoms and can last from 2 weeks to 20 years or more. (c) AIDS defined by low CD4^+^ T cell counts <200/μl, increased viral loads, various infections opportunists and cancers^1^, ^2^

HIV kills CD4^+^ T cells by three mechanisms: (a) by direct viral destruction of infected cells, (b) by increasing apoptosis rates in infected cells, and (c) by CD8 cytotoxic cell‐mediated killing of infected CD4^+^ T cells. When CD4^+^ T‐cell numbers drop below a critical level, cellular immunity is lost and the body becomes progressively more susceptible to opportunistic infections and neoplasms. The stage of infection, which presents different phases, can be determined by measuring the CD4^+^ T‐cell counts and the viral load of the patients (Figure 2).

The transmission of HIV is greatly influenced by the amount of infectious virus particles in a body fluid and the extent of contact with that body fluid. Epidemiological studies during 1981 and 1982 indicated that the main routes of transmission of HIV were intimate sexual contact and contaminated blood. AIDS was initially described in homosexual and bisexual men and intravenous drug users, but its transmission as a result of heterosexual activity was also soon recognized. Furthermore, it became apparent that transfusion recipients and haemophiliacs could contract the disease by transfusion of blood or blood products and that mothers could transfer the causative agent to newborns as well. These three main means of transmission: parenteral, sexual and vertical (including during pregnancy at delivery and through breast milk) can be largely explained by the high concentrations of HIV in various body fluids. It is worth mentioning that the “optimal prevention scenario” would be the pregnant woman adheres to treatment, under regular care and with a suppressed HIV viral load of <50 copies of RNA/ml throughout pregnancy and lactation. When these criteria are met, the theoretical risk of mother‐to‐child transmission is practically zero.^18^, ^19^ Vertical transmission of HIV occurs in 11%–60% of children born to HIV‐positive mothers.^20^ The latest findings suggest that the level of free infectious virus in maternal blood could predict the infection outcome of the newborn.^2^, ^17^ Vertical HIV transmission may decrease from 25% to 2% with the use of ARVs that include nucleoside analogues, during pregnancy. However, there is some evidence that exposure in the womb to nucleoside analogues can cause mitochondrial dysfunction symptoms in a small number of HIV‐uninfected children.^21^ More studies are needed to elucidate the mechanisms of the mitochondrial dysfunction, focusing on exposure in utero, and to identify the importance of mitochondrial variations in children without clinical signs of mitochondrial dysfunction.^21^

#### 2.1.1 HIV structure

HIV is a spherical particle 120 nm in diameter made up of three layers, including (i) a lipid envelope which is an external bilayer of phospholipids, coming from the infected host cell, containing class I and II histocompatibility antigens and some adhesion molecules that facilitate contact with target cells. It also contains 72 copies of a viral glycoprotein complex, called Env that protrudes through the surface to the outer environment. These glycoprotein complexes consist of a head of three gp120 glycoproteins and a body of three gp41 molecules, anchored to the molecules of the viral envelope. They allow the virus to bind and fuse cells to start the infectious cycle. Both surface proteins have been considered as possible targets for future development of treatments or vaccines.^22^ (ii) A capsid or matrix which is a spherical intermediate structure containing p17 protein and (iii) a nucleocapsid or nucleus which is an icosahedral internal structure, consisting of p24 protein. It contains the viral genome (two identical, single‐stranded RNA molecules), the p9 and p7 nucleoproteins, and the machinery required for viral replication (reverse transcriptase, integrase and protease). As a retrovirus, its enzyme, reverse transcriptase, converts viral RNA into proviral DNA. The HIV genome is characterized by a high mutation rate, due to the errors of the reverse transcriptase enzyme during the back transcription of RNA into DNA, and the recombination capacity of the different viruses that can coexist within a cell. Consequently, HIV has high genetic variability, which hinders both the defence response of the immune system and the creation of an effective vaccine system against the virus.^2^

#### 2.1.2 Replication cycle of HIV

Like all obligate intracellular pathogens, HIV must take advantage of the multiple functions of the host cell to replicate successfully.^23^ When HIV enters the target cell, the viral RNA genome becomes double‐stranded proviral DNA and is imported into the cell nucleus and integrated into the cellular DNA by a virally encoded integrase and host cofactors.^24^ Once integrated, the virus can become dormant, allowing both the virus and its host cell to avoid detection by the immune system. Alternatively, the virus can be transcribed, producing new viral RNA and protein genomes that are packaged and released from the cell as new viral particles that begin the replication cycle. Importantly, most of the HIV proteins exert mitochondrial interactions, as depicted in Table 1. Mitochondrial changes derived from the viral interactions occur either in the host cells (mainly CD4^+^ T‐cell lymphocytes and macrophages, but also other lymphocytes, neuronal and glial cells from the central nervous system (CNS), enterochromaffin cells from the gut and dendritic cells, including Langerhans cells,^33^ or bystander cells. Apoptosis of uninfected bystander cells is a key element of HIV pathogenesis and represents a driving force to the important CD4^+^ loss which cannot be explained only by the direct infection.^34^ While several viral proteins have been implicated in this process, the complex interaction between Env glycoprotein expressed on the surface of infected cells and the receptor and co‐receptor expressing bystander cells has been proposed as a major mechanism. Laurent‐Crawford et al.^35^ were the first to demonstrate that the HIV Env glycoprotein alone expressed on the surface of cells is capable of inducing cell death in neighbouring T cells. Importantly, the effects of HIV proteins and/or ARV on mitochondria may differ depending not only on whether the target is a host or a bystander cell, but also on the cell type. As an example of the latter, HIV gp120 and Tat have been shown to alter autophagy and mitophagy in neurons and Tat also alters mitophagy in microglial cells.^36^ Although this could certainly affect children, no data have been reported so far in paediatric population.

**TABLE 1:** Viral proteins of HIV and mitochondrial interactions in the host cells

Type	Protein	Mechanism of action and mitochondrial interactions
Structural	Env^17^	Allows the virus to target and bind to specific cell types and infiltrate the cell membrane Increases Bax (pro‐apoptotic) Decreases Bcl‐2 (anti‐apoptotic) Activates mitochondrial apoptosis
Regulatory	Tat^17^, ^25^, ^26^, ^27^, ^28^	Reduces the expression of the mitochondrial superoxide dismutase 2 isoenzyme, (endogenous inhibitor of the permeability of the mitochondrial membrane) and triggers the loss of mitochondrial membrane potential Increases Fas ligand expression in T cells, inducing apoptosis Promotes Tat secretion by infected cells, promoting mitochondrial apoptosis in uninfected T cells Induces apoptosis by a mechanism involving disruption of calcium homeostasis
	Rev^17^	Ensures the replication of HIV in the infected cell Targets the permeability transition pore, allowing the permeabilization of the mitochondrial membranes
Complementary	Nef^17^	Regulates CD4^+^ expression on the cell surface Disrupts T cell activation Stimulates HIV infectivity
	Vpr^29^, ^30^, ^31^, ^32^	Blocks the cell cycle in G2 Blocks cell division Prevents the activation of the complex p34cdc2/cyclin B, a known cell cycle regulator, required for entering into mitosis Regulates apoptosis and transcriptional modulation of immune function
	Vpu^29^	Promotes CD4^+^ modulation Increases the release of virions Is responsible for releasing the viral envelope, triggering the degradation of CD4^+^ molecules bound with Env

The viral cycle is divided into four stages: (i) fusion and entry of HIV: The gp120 glycoprotein binds CD4, undergoes a conformational change, and interacts with the cell co‐receptor (CCR5 or CXCR4), prompting conformational changes in the viral gp41 glycoprotein.^37^ Subsequently, once fused with the cell membrane, HIV releases its genetic material (viral RNA) into the cytoplasm of the cell, along with viral proteins.^2^ (ii) Reverse transcription and integration of proviral DNA: single‐stranded RNA is converted to double‐stranded DNA through the activity of the viral reverse transcriptase enzyme. Viral proteins help double‐stranded proviral DNA reach the nucleus and integrate into the cell genome, through the virus integrase enzyme. In the event of HIV entering a quiescent cell, the proviral DNA will accumulate in the cytoplasm without any integration, leading to latency. The latent provirus that exists as a reservoir within quiescent cells greatly hampers both the effective endogenous system and HIV treatment, as it avoids immune and exogenous control.^2^ (iii) Expression of the viral genome: once the proviral DNA has been integrated into the target cell nuclear genome, some viral proteins, along with cellular transcription factors, such as nuclear factor kappa, enhancer of the B‐cell light chain activated κB (NF‐κB), induce replication and transcription of the viral genome. Initially, transcription leads to the synthesis of HIV regulatory proteins (Tat, Rev, Vpr, Vpu and Nef).^17^, ^25^, ^26^, ^27^, ^29^, ^30^, ^31^, ^32^, ^38^ Messenger RNA is produced as a single transcript that is transported to the cytoplasm and is processed into many different RNAs of different sizes. HIV protease is in charge of the conversion of a large protein precursor molecule to small active and functional molecules.^2^, ^24^ (iv) Assembly of new viral particles: all functional viral compounds are assembled giving rise to new viral particles that are released into the bloodstream, to infect other cells. The lifespan of HIV in plasma is 6 h. To maintain a constant viral concentration in the body,^39^, ^40^ new viral particles are produced daily. This fact makes it difficult to find an effective treatment against the virus.^2^

#### 2.1.3 HIV in the paediatric population

The development of effective therapy for HIV infection has substantially reduced HIV‐related morbidity and mortality, making HIV infection a chronic disease.^41^ The life expectancy of people with HIV has increased in countries where ARVs are widely used, although the continued spread of the pandemic has increased the number of people living with HIV. In 2018, around 1.7 million people contracted HIV globally, a 16% drop from 2010 that is driven, mostly, by steady progress in most of Eastern and Southern Africa. For example, South Africa has come a long way as it has significantly reduced new HIV infections (by more than 40%) and AIDS‐related deaths (by approximately 40%) since 2010.^42^ In 2018, an estimated 37.9 million (32.7 million–44.0 million) people were living with HIV: 36.2 million (31.3 million–42.0 million) adults and 1.7 million (1.3 million–2.2 million) children (under the age of 15). Sixty‐two percent (47%–75%) of adults over the age of 15 living with HIV had access to treatment, as did 54% (37%–73%) of children up to 14 years old. Importantly, since 2010, new HIV infections in children have decreased by 41%, from 280,000 (190,000–430,000) in 2010 to 160,000 (110,000–260,000) in 2018.^42^

In most cases, the diagnosis of vertical transmission of HIV is made in the first weeks of life: the viral genome is detected by polymerase chain reaction (PCR) in 93% of infected newborns at 15 days of life. The sensitivity and specificity of these tests increase to 96%–99% at the age of 1 month.^43^ Earlier diagnosis allows rapid implementation of ARV treatment in the acute stage of infection. In patients who have acquired HIV infection by vertical transmission, acute infection is not associated with the acute retroviral syndrome that occurs in 60% of newly infected adults.

As mentioned previously, HIV replicates in CD4^+^ T cells and progressively destroys the immune system. In children, since the immune system is not fully developed, immune suppression as well as AIDS develops faster than in adults. Consequently, in the first years of life, viral loads remain very high in plasma in the absence of ARV. The first symptoms of vertical HIV infection are usually nonspecific and develop during the first year of life. Opportunistic infections present in patients with severe immune suppression and, in most cases, have a worse evolution than in adults (such as pneumonia caused by *Pneumocystis jirovecii*).^2^

After vertical transmission, there are mainly two evolutionary patterns of progression of HIV infection: fast progressors (30%) and slow progressors (65%). Clinical manifestations during the first months of life will determine the prognosis. For example, HIV‐associated encephalopathy and pneumonia caused by *P. jirovecii* are predictors of rapid progression, while chronic parotitis or lymphoid interstitial pneumonia is associated with slow progression. A third group of children (<5%) has also been described: very slow progressors, who remain with normal CD4^+^ T‐cell counts and low viral loads for years, without any treatment.

#### 2.1.4 Mitochondrial changes in HIV infection

Mitochondrial impairment was first associated with HIV in the 1990s,^44^ and in 2002, mtDNA depletion (a decrease in mtDNA copies) was described in mononuclear cells in the peripheral blood of HIV‐infected patients who had never received ARV.^45^

HIV causes mitochondrial impairment by triggering apoptosis; many viral proteins are known to have the ability to induce apoptosis, as already mentioned above.^46^ HIV infection produces an increase in the levels of tumour necrosis factor α (TNFα), a cytokine produced in most inflammatory and immunological reactions, which is an apoptotic inducer. It occurs in lymphocytes as an anti‐HIV response, and it also promotes HIV replication in T cells through activation of NF‐κB transcription.^47^ In general, HIV‐derived apoptosis affects infected and uninfected CD4^+^ T cells, contributing to leukopenia, typical of infected patients.^48^

Furthermore, and partly as a result of increased apoptosis, HIV‐infected cells show an imbalance between oxidants and antioxidants.

Another HIV‐associated toxic effect is Ca^2+^ overload and activation of nitric oxide synthase (NOS). This enzyme, which catalyses the formation of nitric oxide (NO) from L‐arginine, can be expressed in neurons (nNOS or NOS‐1), as well as by activated microglia (iNOS or NOS‐2). Increases in NO can react with cellular superoxide to form peroxynitrite and promote various forms of neurodegenerative diseases.^49^ Tat affects both iNOS and nNOS, increasing Ca^2+^ by releasing intracellular deposits as well as through Ca^2+^ entry, induced by activation of N‐methyl‐D‐aspartic acid (NMDA) receptors. The toxic effects of Ca^2+^‐induced increases in Tat are mitigated by Ca^2+^ chelators, as well as inhibitors of Ca^2+^ absorption in mitochondria,^28^ supporting the role of Ca^2+^ dysregulation and Tat neurotoxicity. In addition to the Ca^2+^ channels of the plasma membrane, eukaryotic cells control Ca^2+^ homeostasis through Ca^2+^ channels, located in the ER, mitochondria and other organelles, through Ca^2+^ buffering proteins, and systems for extrusion and sequestration of Ca^2+^.^50^ Therefore, it is important to consider that Tat may also affect Ca^2+^ homeostasis in a manner independent of the NMDA receptor. In fact, Tat depletes both mitochondrial and ER Ca^2+^ by activating ryanodine receptors.^51^ Furthermore, Tat appears to increase Ca^2+^ by activating L‐type channels.^52^ Thus, Tat appears to disrupt Ca^2+^ homeostasis by affecting both ER and other Ca^2+^‐controlling organelles and Ca^2+^ regulatory systems located in the plasma membrane.^53^ Regardless of the mechanisms, all evidence points to altered Ca^2+^ homeostasis as one of the main mechanisms of Tat neurotoxicity. It should be mentioned that viral gp120 appears to modulate Ca^2+^ by a different mechanism. In fact, in contrast to Tat, gp120 increases Ca^2+^ mainly by mobilizing calcium deposits sensitive to inositol triphosphate (IP3).^54^ Because viral protein‐induced mitochondrial toxicity has been repeatedly associated with disruption of Ca^2+^ homeostasis, it is not surprising that indirect ways to prevent Tat or gp120 toxicity include receptor‐mediated blocking of Ca^2+^ entry. This includes the reduction of NMDA receptor activation by mild receptor antagonists, such as memantine, which protects neuronal function against gp120‐mediated toxicity.^55^

NO has antiviral effects and increases within the cell in the presence of HIV, however, NO and ONOO^−^ contribute to oxidative damage to cells and direct inhibition of mitochondrial respiration.^56^

Since changes in Ca2+ homeostasis, some of them above explained, have an influence in mitochondrial dynamics.^57^ Mitochondrial dynamics are also affected by HIV. Mitofusin 1 (Mfn1) and mitofusin 2 (Mfn2) are required to promote fusion of two neighbouring mitochondria. In contrast, mitochondrial fission is mediated by dynamin‐related protein 1 (Drp1), which divides a mitochondrion into two. Fission helps by splitting healthy from defective mitochondria. Damaged mitochondria are then recycled or degraded through mitophagy; otherwise, apoptosis begins. In fact, both Tat and gp120 from HIV promote mitochondrial fragmentation (fission) and mitophagy alterations in human neurons.^58^

The mitochondrial dynamics of fusion and fission, estimated by Mfn2/β‐actin and Drp1/β‐actin contents, are decreased in the placenta of HIV‐infected pregnant women, although there is a lack of information as to whether the newborn continues to present such alterations or not.^59^

Several HIV proteins activate key components of the transient permeability transition pore (PTP), leading to mitochondrial membrane depolarization. Furthermore, Tat can cause the translocation of Bim, a member of the pro‐apoptotic family Bcl‐2, from microtubules to mitochondria, where it induces PTP. Acute Ca^2+^ overload caused by Tat can also trigger the formation of PTP complexes. Furthermore, this protein can promote mitochondrially induced apoptosis.^60^

The use of ARVs minimizes HIV‐related mitochondrial deterioration by decreasing viral load to undetectable levels. However, ARVs are also linked to side effects, as described in the following section. Therefore, mitochondrial toxicity is ultimately determined by both viral load and ARV exposure. In clinical practice, it is often difficult to differentiate whether mitochondrial abnormalities are related to HIV itself, or to ARVs.^2^

#### 2.1.5 ARV treatment in the paediatric population and mitochondrial involvement

**FIGURE 3:**
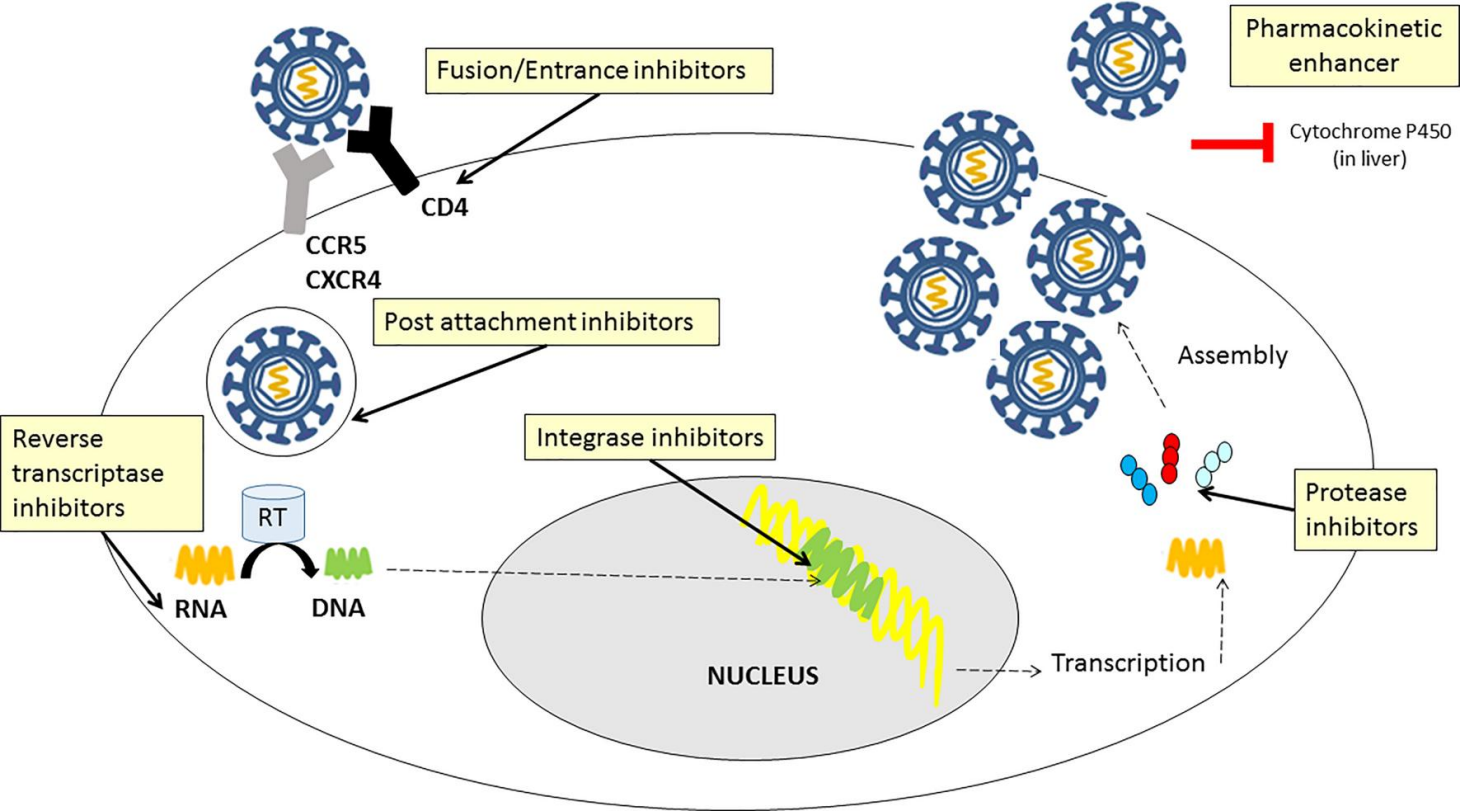
Site of action of the different types of antiretroviral treatment within the host cell during HIV replication. Fusion and entrance inhibitors block the fusion and entrance of the virus in the host cell. Reverse transcriptase inhibitors block the retrotranscription from viral RNA to DNA. Integrase inhibitors inhibit the integration of proviral DNA into the cell nuclear genome. Protease inhibitors block the protease enzyme and therefore the assembly of the virions. Post‐attachment inhibitors block the HIV from attaching the CCR5 and CXCR4 co‐receptors of the host cell

The different families of ARVs and the site of action are shown (Figure 3). Currently, ARV implementation has dramatically improved mortality and morbidity from HIV infection by decreasing viral load to undetectable levels and increasing CD4^+^ T‐cell counts. In addition, simplification of therapeutic administration has led to better adherence to therapy. In developed countries, due to ARV administration, HIV infection is considered a chronic disease rather than a lethal infection.^2^ Importantly, several anti‐HIV drugs may also lead to mitochondrial alterations at different levels, which has been summarized, including paediatric studies (Table 2).

**TABLE 2:** HIV antiretroviral agents and derived mitochondrial dysfunction including paediatric studies

Antiretroviral family	Characteristics	Mechanism of action	Mitochondrial dysfunction	Clinical secondary effects	Paediatric studies
Nucleoside/nucleotide reverse transcriptase inhibitors (NRTI), e.g., ABC, FTC, 3TC, TDF and ZDV^2^, ^10^, ^61^	Antagonists of natural nucleosides: adenine, thymine, cytosine and guanine Adequate resistance profile Excellent tolerability High bioavailability Once daily treatment (except for ZDV)	Interfere with reverse transcriptase protein of HIV, which is necessary for viral replication	Inhibition of mtDNA polymerase gamma mtDNA depletion (a) by means of a direct inhibition of DNA polymerase (b) By inducing errors during replication (c) By reducing exonuclease repair capacity Decrease of mtDNA encoded proteins General dysfunction of MRC Direct inhibition of complexes of MRC (I–IV) Decreased levels of ATP ROS production Decrease of mitochondrial membrane potential Δψm Impairment of ADP/ATP translocase Impairment of fatty acid oxidation^62^, ^63^, ^64^, ^65^ NAD+/NADH impairment Increased apoptosis Overexpression of the Fas receptor^66^	Lactic acidosis, polyneuropathy, pancreatitis or lipodystrophy, among others	Complex and multifactorial mechanism: Genetic predisposition, dose and type of NRTI and duration of exposure ZDV increases the risk of decreased blood mtDNA content which may be associated with altered mitochondrial fuel in infants^67^
Non‐nucleoside reverse transcriptase inhibitors (NNRTI) e.g., EFV, ETR, NVP, RPV and DOR^2^, ^10^, ^61^	They do not need to compete with natural nucleosides They are activated within the cell, directly interacting with viral reverse transcriptase and blocking its activity^2^, ^68^, ^69^	Stops HIV replication within cells by inhibiting the reverse transcriptase protein of HIV	Mitochondrial dysfunction through bioenergetics stress (e.g., EFV has been associated to alterations in MRC in cultured glial cells and neurons^70^	NVP and EFV have been associated with hepatotoxicity	In an urban area of Togo, the resistance of children with HIV type 1 treated with two NRTIs and one NNRTI showed mutations related to NNRTI class, with 100% mutations for EFV and NVP. The need to use PI is shown in most children treated with NNRTI^71^, ^72^, ^73^
Protease inhibitors (PI) e.g. LPV/rtv, *ATV/rtv, DRV/rtv* ^2^, ^10^, ^61^	Block maturation and activation of viral proteins (in an advanced stage of the viral cycle) Metabolization by cytochrome P450, therefore, pharmacokinetic interaction with other drugs is common	Inhibit protease activity of HIV, a protein required for viral replication	Mitochondrial network fragmentation Mitochondrial Ca^2+^ accumulation Apoptosis ROS production Alterations of glucose and lipid metabolism^2^, ^74^	Peripheral neuropathy,^75^ lipodystrophy, metabolic syndrome, insulin resistance, diabetes, or cardiovascular risk	Some studies report low tolerability, problems of adherence and development of resistance to treatment in children^76^
Integrase inhibitors (II) e.g., RAL, DTG and EVG^2^, ^10^, ^61^	Inhibit the integration of the viral genome into the nuclear genome of the cell	Interfere with the viral enzyme integrase, which is needed to insert HIV genetic material into genetic material of human cells	Expected cytotoxicity is low for most of them, as they suppress the viral cycle at very early stages	Severe skin reactions, allergic reactions and liver disorders	WHO recommends regimens based on DTG, once formulations suitable for children are widely implemented and available, as well as ongoing dosage and safety studies are completed; this will significantly ameliorate treatment outcomes^76^
Fusion inhibitors (FI) e.g., *T‐20* ^2^, ^10^, ^61^	Block the fusion between HIV membrane and the target cell Limited effectiveness	Prevent the virus from binding to human immune cells		Slight reaction in the area of application. Possible nausea, diarrhoea, vomits, headache and insomnia	The pharmacokinetic profile in children and adolescents with HIV infection is similar to that in adults^77^ T‐20 pharmacokinetics in children were not affected by age, bodyweight, body surface area or puberty stage^78^ In paediatric patients, efficient HIV‐1 replication control is limited by their immature immune system^79^
Entrance inhibitors (EI) e.g., *MVC* ^2^, ^10^, ^61^	Block the entrance of the virus into the host cell by inhibiting CD4^+^ T‐cell receptors or CCR5 co‐receptors, and promote a conformational change, where the virus needs to be anchored			They present a very favourable safety profile^80^ In the MERIT study MVC caused insignificant changes in total cholesterol, low‐density lipoprotein, high‐density lipoprotein and triglycerides^81^	The effects of EI drugs are considered sufficiently similar in paediatric and adult patients to allow for extrapolation of efficacy data^82^ MVC is well tolerated^82^
Pharmacokinetic enhancer, e.g. *COBI* ^2^, ^10^, ^61^	They are used in combination with a primary ARV agent (either a PI or EVG), not for their direct effects on HIV replication, but because they enhance the activity, increase drug levels and/or prolong the half‐life of the primary agent^83^	Inhibitor of CYP3A4 that increases systemic exposition of the primary agent	N/A	Jaundice, diarrhoea, cephalea, rash or nausea	Safe and effective in paediatrics A study in pregnant women has shown less exposure to EVG and COBI during the second and third trimesters of pregnancy compared to the postpartum period. This could lead to virological failure and an increased risk of transmission of HIV infection from mother to child^84^

*Note*: There is another family of ARV, the post‐attachment inhibitors, such as ibalizumab‐uiyk (IBA), which are not approved in children, but in the next coming future may be considered as an option in the paediatric population.

Abbreviations: 3TC, lamivudine; ABC, abacavir; ARV, antiretrovirals; ATV, atazanavir; COBI, cobicistat; DOR, doravirine; DRV, darunavir; DTG, dolutegravir; EFV, efavirenz; ETR, etravirine; EVG, elvitegravir; FPV, fosamprenavir; FTC, emtricitabine; MRC, mitochondrial respiratory chain; mtDNA, mitochondrial DNA; MVC, CCR5 antagonist‐ Maraviroc; NVP, nevirapine; RAL, raltegravir; ROS, reactive oxygen species; RPV, rilpivirine; RTV, ritonavir; SQV, saquinavir; T‐20, enfuvirtide; TDF, tenofovir; TPV, tipranavir; ZDV, zidovudine; Δψm, mitochondrial transmembrane potential.

#### 2.1.6 Interactions between ARVs and mitochondria

The use of ARVs has dramatically reduced the mortality and morbidity associated with HIV infection and AIDS, keeping the viral load undetectable and CD4^+^ T‐cell counts within normal values. However, this treatment has been associated with many side effects, such as allergies, hypersensitivity to nucleoside reverse transcriptase inhibitor (NRTI), hepatotoxicity, fever, malaise, gastrointestinal disturbances, anaemia, leukopenia, hyperpigmentation of the skin, insulin resistance or diabetes mellitus, renal disorders, decreased bone mass, myopathy, hyperlactatemia and lactic acidosis, pancreatitis, peripheral neuropathy, and disorders of the CNS, such as depression, mood changes or insomnia, among others.^2^ Most clinical adverse events are undoubtedly related to mitochondrial abnormalities. In fact, mitochondrial deficiencies may partially explain the aetiopathogenesis of most ARV‐related clinical manifestations which have been documented, including paediatric studies (Table 3).

**TABLE 3:** Clinical toxicity of the NRTI

NRTI‐derived clinical secondary events	Monitoring biomarkers and altered clinical parameters	Mitochondrial events	Paediatric studies in exposed and/or infected children
Haematological toxicity	Anaemia Neutropenia Thrombocytopenia^85^ Permeation of the drugs into canine bone marrow progenitor cells^60^	MtDNA depletion, mutations and MRC dysfunction in peripheral blood mononuclear cells^86^	At 0–2 months of age: Haemoglobin concentrations, neutrophil, lymphocyte and CD4+ Cell counts are lower At 6–24 months of age: Differences in platelet, lymphocyte and CD4^+^ cell counts persisted and CD8^+^ cell counts became significantly loweriii. In comparison with ARV monotherapy, combination therapy was associated with larger decreases in neutrophil, lymphocyte and CD8^+^ cell counts at age 0–2 months but with differences only in CD8^+^ cell counts at 6–24 months^87^ MtDNA depletion and MRC dysfunction in peripheral blood mononuclear cells from infants has been reported^65^, ^88^, ^89^
Cardiomyopathy	Ultrastructural changes in cardiomyocytes^60^	Increased lactate production derived from mitochondrial dysfunction and decreased activities of respiratory chain CII and CIV in myocytes from human muscle, with ddC being the most toxic agent^90^	Findings of multifactorial origin (including mitochondrial alterations): Increased global risk of premature cardiovascular disease in perinatally HIV‐infected children and adolescents Increased carotid intima‐media thickness and arterial stiffness in HIV‐infected children and adolescents Elevated metabolic and inflammatory markers of atherosclerotic disease^91^
Neuropathy	Peripheral neuropathy. Distal symmetric polyneuropathy Inflammatory demyelinating polyneuropathy Mononeuritis multiplex Progressive polyradiculopathy Autonomic neuropathy^60^ In vitro evidence of neuronal and glial damage^92^	DdC, ddI and d4T (not currently used) inhibit mitochondrial membrane potential directly to cause neurotoxicity in dorsal root ganglion neurons Impairment of Ca^2+^ signalling pathways Reactive oxygen species Apoptosis^93^	Children exposed to nucleoside analogues during the perinatal period are at risk of a neurological syndrome associated with persistent mitochondrial dysfunction.^94^ This is supported by findings observing the capacity of some ARV trespassing the blood–brain barrier and promoting mitochondrial damage in the brain^95^ Distal sensory polyneuropathy is a potential problem in children on d4T‐based ARV^96^ HIV infection affects central nervous system structures mediating motor and spatial memory development, even in asymptomatic children.^97^ This is also supported by in vitro studies observing oxidative stress induction and neuronal damage derived from ARV in CNS^92^
Pancreatitis	Immunodeficiency Elevations of amylase and lipase	Disruption of Ca2^+^ homeostasis causes mitochondrial dysfunction and pancreatic damage^98^	Early paediatric studies described cases in children receiving 3TC^60^ Acute pancreatitis has never been reported as a presenting manifestation of acute HIV infection in children Pancreatitis is uncommon in children and adolescents, and the causes are more varied than in adults^99^
Lactic acidosis	Increased lactate levels in serum Seldom manifesting as acute lactic acidosis with evidence of hepatic steatosis probably the most worrisome toxicity (although this is not currently observed, since the most toxic ARVs are not being used)	Overproduction of lactate derived from mitochondrial damage^100^ Insufficient oxidative phosphorylation	In utero and perinatal exposure to NRTI trigger hyperlactatemia from mitochondrial toxicity^101^, ^102^ The clinical presentation of lactic acidosis is unspecific in children and may include gastrointestinal symptoms (nausea and vomiting, abdominal pain)^91^ Chronic symptom‐free hyperlactatemia has been reported in up to one‐third of HIV‐infected children Symptomatic hyperlactatemia with or without lactic acidosis has been reported in children^91^ Sporadic cases of lactic acidosis have been reported with all available NRTI, but exposure to d4T and ddI is associated with the highest risk, especially when the two drugs are used together^91^
Lipodystrophy	Acidemia and peripheral fat wasting Three main types: Lipohypertrophy Lipoatrophy (especially related to d4T and ZDV) Mixed pattern Reduction in plasma lactate levels as lipodystrophy improves^60^	MtDNA depletion Mitochondrial ultrastructural abnormalities in the mitochondria^103^	HIV‐infected children showed lower mtDNA levels and a reduction in global mitochondrial CI‐CIII‐CIV enzymatic activity, which was more pronounced in HIV‐infected children presenting lipodystrophy compared to asymptomatic children^104^ Pubertal development, older age and longer time on HAART have been identified as risk factors for lipohypertrophy^91^
Renal toxicity	Concentration in the cells of the proximal tubule	Prevalent in the HIV‐infected paediatric population, due to the increasing use of TDF It is uncertain how commonly, and how long after TDF implementation, renal toxicity occurs in HIV‐infected paediatric patients^91^ Inhibition of mtDNA polymerase Decompensated hyperlactatemia derived from mitochondrial failure^60^ However, mild tubular dysfunction is recognized in a substantial proportion of TDF‐treated individuals and tends to increase with cumulative exposure^105^	Severe renal damage associated with TDF use is uncommon and of multifactorial origin in children The median blood urea nitrogen increases for every 6‐month increment in ARV duration in a cohort of children^106^
Myopathy	Myalgia	Red‐ragged fibres’ Abnormalities in mitochondrial morphology^60^ Muscular mitochondrial dysfunction as shown by rapid increases in lactate level Impairment of respiratory chain activity for CIII and CIV Mitochondrial histoenzymatic abnormalities^107^	Myoblasts can differentiate into myotubes and are more abundant in the skeletal muscle of infants and children than in adults. Moreover, age is known to alter the potential of myoblasts to differentiate into myotubes and to affect myoblast metabolism and proliferation. These differences are of particular interest, because the decline in mtDNA resulting from ddI exposure has been found greater for myoblasts than myotubes The effects of each NRTI on mitophagy may, in part, determine the degree of mtDNA and mtRNA degradation^108^
Hepatic toxicity	From mild hepatic abnormalities, to a rare life‐threatening condition with lactic acidosis and hepatic insufficiency Liver histology shows massive steatosis^109^	Inhibition of the DNA polymerase gamma leading to mtDNA mutations and oxidative stress^109^	In a study including 705 children <18 years old, 25.1% presented an elevated AST level, and 11.8% presented an elevated ALT level. Children with elevated AST were younger and were more likely to be on a ZDV‐ or NVP‐based regimen Normalization of liver enzymes was observed during the follow‐up^106^

Abbreviations: 3TC, lamivudine; ALT, alanine aminotransferase; ARV, antiretroviraL; AST, aspartate aminotransferase; CII, complex II; CIII, complex III; CIV, complex IV; d4T, stavudine; ddC, zalcitabine; ddI, didanosine; MRC, mitochondrial respiratory chain; mtDNA, mitochondrial DNA; mtRNA, mitochondrial RNA; NRTI, nucleoside Reverse transcriptase inhibitor; NVP, neviparine; TDF, tenofovir; ZDV, zidovudine.

#### 2.1.7 Paediatric studies of mitochondrial interaction in HIV infection

Mitochondrial abnormalities can lead to metabolic complications in HIV‐infected children who have been receiving long‐term ARV treatment.^110^ Mitochondria can directly influence the infectivity of HIV, the course of HIV infection and the prevalence of side effects of primary therapy.^39^

ARV agents are generally considered safe, although they have been associated with mitochondrial toxicity in experimental and clinical studies. To date, the main focus of ARV‐related mitochondrial toxicity research has been the effects of NRTI on OXPHOS by inhibiting mitochondrial gamma polymerase, the only enzyme responsible for the replication of mtDNA^21^; and, accordingly, our group found CIV enzymatic activity of the MRC was consistently lower in HIV/ARV‐exposed children when compared to healthy controls over time, at 6 weeks and 3, 6 and 12 months of age, with a linear trend toward normalization with age.^40^ A global MRC CI + CIII + CIV enzymatic activity in HIV‐infected mothers and their infants was also observed by our group,^88^ and we found homeostatic‐compensatory mechanisms at the transcription level.^111^

There is evidence that exposure to NRTI in utero and the neonatal period may cause lactic acidosis and a decrease in the number and function of mitochondria that may persist in the child, potentially affecting the growth and development of this otherwise healthy infant. Placental tissue of HIV‐1‐infected ARV‐exposed pregnancies shows evidence of mtDNA depletion with secondary respiratory chain compromise.^112^ Infants exposed to long‐term ARV therapy are more likely than infants not exposed to ARVs to have fatty acid oxidation dysfunction as measured by acylcarnitine analysis. Thus, ARVs may adversely affect intermediate energy metabolism, particularly fatty acid oxidation, suggesting a mechanism of generalized mitochondrial dysfunction, likely due to OXPHOS disruption induced by NRTI.^113^ The use of ARV drugs in human pregnancy is one of the most successful strategies to fight against HIV/AIDS, as it protects thousands of children worldwide from HIV infection. However, there are an increasing number of HIV‐uninfected children that were exposed in utero to HIV and ARVs. Children exposed to HIV in utero generally function without problems, although some clinical studies and evidence from several biomarkers suggest that there may be progressive changes that will compromise important organs, such as the heart and brain, as ageing occurs.^113^

As previously commented, in addition to infection and depletion of T cells, HIV rapidly enters the CNS where it productively infects macrophages, microglia and to some extent, astrocytes.^114^ In fact, mitochondrial dysfunction has been claimed to potentially be a common pathway in HIV‐associated neurological disorders and therefore a promising therapeutic target.^36^ The presence of the virus leads to progressive cognitive disturbances in a large subset of infected individuals. Even with early ARV therapy, more than 50% of HIV patients in the United States develop HIV‐associated neurocognitive disorders ranging from asymptomatic to severe dementia. From a behavioural point of view, HIV‐associated neurocognitive disorder (TNAV) is characterized by executive dysfunction and memory problems, with significant problems in attention, multitasking and judgement, as well as memory encoding and retrieval. One of the distinctive neuropathological features that most correlates with these cognitive deficits in synaptodendritic damage, particularly decreased synaptic and dendritic density.^55^, ^115^ In neurocognitive disorders, neurons experience synaptodendritic abnormalities and damage that can lead to cell death. A strong correlation factor for TNAV is believed to be oxidative stress. Because mitochondria are the main source of ROS responsible for oxidative stress, mitochondrial abnormalities probably have a considerable contribution to the pathogenesis of these disorders.^39^, ^116^ Due to all these events, children may be more vulnerable than adults to the adverse effects of ARVs due to the potential negative impact on growth and development with their long‐term exposure. However, information on the frequency and severity of long‐term adverse effects in children is limited.^89^ Periodic surveillance studies and molecular epidemiology are required in long‐term pretreated HIV‐infected paediatric populations to optimize treatment regimens and to better understand the long‐term dynamics of viral resistance and variants of HIV.^117^

### 2.2 Human cytomegalovirus

HCMV is a virus of global distribution, with seroprevalence rates ranging from 50% to 100% in different world regions.^118^ In immunocompetent individuals, the primary infection is usually asymptomatic, or causes a mononucleosis syndrome. The virus remains latent thereafter in monocytes (including CD34^+^ progenitor cells) and possibly also in other organs and tissues. HCMV can cause recurrent infections either by reinfection with another strain or by reactivation of the latent strain. HCMV cellular tropism is diverse and includes epithelial, endothelial, fibroblast and most immune cell types.^119^, ^120^

HCMV is excreted in urine, saliva, vaginal secretions, semen and breast milk. Primary infection occurs after direct contact with such fluids from an infected person (horizontal transmission), or during pregnancy, childbirth, or breastfeeding (vertical transmission). In immunocompetent adults, viral excretion is intermittent and indefinite, while in immunosuppressed patients and children with congenital, perinatal, or early postnatal infection, viral excretion is prolonged (even for years) and constant.^121^

#### 2.2.1 Structure and replication cycle

The structure of the HCMV virion consists of the nucleocapsid containing linear double‐stranded DNA, a capsid protein with 162 capsomeres, another protein layer called tegument, which contains phosphoproteins, and a lipid envelope into which viral glycoproteins are inserted, acting as mediators for the entry of the virus into the host cell.

**FIGURE 4:**
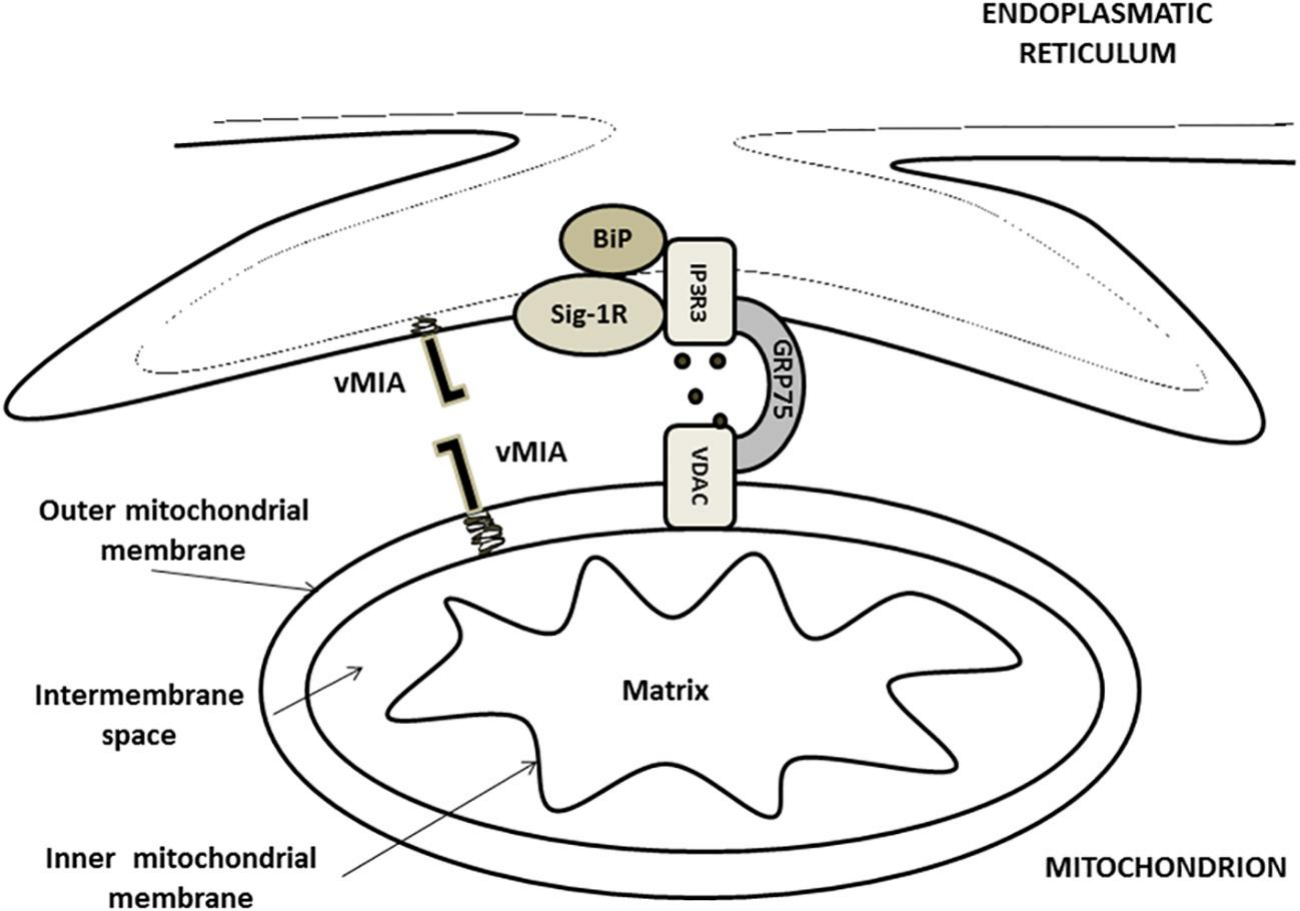
Mitochondria‐associated membranes or MAM: endoplasmic reticulum and mitochondrial sub‐compartments. Contact is shown with IP3R3, a Ca^2+^ signalling complex components on the ER; GRP75 on cytosol and VDAC on the outer mitochondrial membrane. Ca^2+^ efflux from ER is regulated by chaperones (BiP and Sig‐1R) as well as vMIA. ER, endoplasmic reticulum; vMIA, viral mitochondria‐localized inhibitor of apoptosis

HCMV proteins are trafficked from the ER into mitochondria, probably through the mitochondria‐associated membrane (MAM) compartment. The MAMs are sites of ER–mitochondrial contact that enables the direct transfer of membrane‐bound lipids and the generation of high Ca^2+^ microdomains for mitochondrial signalling and responses to cellular stress (Figure 4). Trafficking of viral proteins to the MAM may allow viruses to manipulate a variety of fundamental cellular processes, which converge at the MAM, including Ca^2+^ signalling, lipid synthesis and transfer, bioenergetics, metabolic flow and apoptosis.^122^

The replication of HCMV is associated with the sequential expression of three gene classes: the immediate early genes, early genes and late genes (Table 4).

**TABLE 4:** Sequential expression of HCMV genes. The genome is expressed as a cascade giving rise to the formation of complete viral particles^120^

Phase 1	Phase 2	Phase 3
i) Enumerations of this Table have problems in all cases **Regulatory** viral α proteins are synthesized. They have regulatory activity over the replication and transcription of early immediate genes	i) Enzymatic viral β proteins are synthesized. They present enzymatic regulatory function in DNA replication	i) Structural viral γ proteins are synthesized. These are the structural proteins of the viron:
ii) Immediate genes: - Take control of the cellular synthesis of macromolecules - Facilitate the expression of early genes	ii) Early genes: - Control the production of virions - Stimulate the transcription of the structural components of the virion, that is, of the late genes	- Glycoproteins involved in the production of neutralizing antibodies - The capsid proteins - The proteins of the integument, phosphoproteins, among which pp65 (ppUL83) stands out, the main target for production of monoclonal antibodies used in disgnostic tests

#### 2.2.2 HCMV in the paediatric population

HCMV infection can lead to intrauterine foetal infection and congenital disease during pregnancy. Congenital HCMV is the most common congenital infection, affecting between 0.2% and 6.1% of all newborns, depending on the seroprevalence in the population.

Congenital HCMV infection occurs commonly among infants born to mothers who had primary HCMV infection during pregnancy. In this situation, transmission of infection occurs in approximately 40% of cases. The risk of transmission is the highest at the end of gestation (65%–70% in the third trimester). Less commonly, transmission of HCMV may occur among infants born to HCMV‐immune women, upon HCMV reactivation or superinfection with a different strain of HCMV, since preconceptional immunity provides only partial protection. However, the abundance of seropositive women means that they deliver most babies with congenital HCMV.^123^ Congenital HCMV infection may manifest as clinical disease at birth in only about 10%–15% of cases. The clinical manifestations can be mild, nonspecific findings to severe, multiple‐organ system involvement. This infection is the leading cause of non‐hereditary hearing loss and severe neurodevelopmental disorders (cerebral palsy, mental retardation, seizures and impaired vision) in newborns. Congenital HCMV disease may present with petechiae (54%–76%), jaundice (38%–67%), hepatosplenomegaly (39%–60%), low weight (39%–50%), microcephaly (36%–53%), hearing loss (34%), prematurity (25%–35%), lethargy‐hypotonia (27%), chorioretinitis (11%–14%), among others. Between 8% and 10% of the newborns can present severe forms of infection associated with a high mortality rate (30%); these are more common in premature infants and newborns with congenital immunodeficiencies. About 10%–15% of infants with congenital HCMV disease may manifest solely with sensorineural hearing loss^124^ that may develop during the first years of life.

Since currently no systematic screening of this infection during pregnancy is recommended, and it is generally asymptomatic, a high degree of clinical suspicion is necessary to identify pregnant women with acute infection and affected foetuses. Most obstetric units rule out HCMV infection in those women with a clinical viral infection, and/or when alterations are detected in the development of the foetus, especially intrauterine growth restriction, microcephaly, or other alterations in the ultrasound results. Upon suspicion of infection in the pregnant woman, the infection can be confirmed by detecting the HCMV genome, using molecular biology techniques in amniotic fluid.^125^ The identification of HCMV‐infected newborns is determined by the suspicion or confirmation of maternal infection, due to the presence of clinical manifestations or after selective screening of those newborns with abnormal audiological testing. Even though the universal screening of HCMV infection in newborns is predicted to be cost effective, it is not performed systematically in most centres.^121^, ^126^

#### 2.2.3 Mitochondrial changes in HCMV infection

Cell death or apoptosis is of utmost importance during the development, morphogenesis and maintenance of homeostasis.^120^ Apoptosis is an important antiviral defence mechanism which is manipulable by various viruses, including HCMV. The effect of HCMV is predominantly considered anti‐apoptotic, but it seems to be dependent on multiple factors such as the viral strain used, the post‐infection time, the viral load and the infected cell type.^120^

Of note, infection with HCMV profoundly affects cellular metabolism. Like in tumour cells, HCMV infection increases glycolysis and glucose carbon is shifted from TCA to the biosynthesis of fatty acids. However, unlike in many tumour cells, where aerobic glycolysis is accompanied by suppression of mitochondrial OXPHOS, HCMV induces mitochondrial biogenesis and respiration, to facilitate its own replication.^127^

In general, following HCMV infection, the MRC functions at an elevated rate releasing increased ROS. Surprisingly, despite the stress applied to the host mitochondria, the network is capable of responding to and meeting the increased bioenergetic and biosynthetic demands placed on it. Importantly, when mtDNA is depleted from the cells, a severe impairment of viral replication is observed.^120^, ^127^

#### 2.2.4 Pro‐apoptotic effects of HCMV

HCMV exerts a direct role disrupting the mitochondrial membrane potential (Δψm); it can be disrupted by transmitting a death signal to mitochondria upon infection of cells by HCMV. CytC is discharged into the cytoplasm. Then, caspase‐3 is activated by combining it with caspase‐9. In addition, caspase‐8 is activated by caspase‐3. Thus, HCMV‐induced apoptosis takes place via an intrinsic mitochondrial pathway.^128^ In fact, HCMV induces apoptosis in neural stem/progenitor cells derived from induced pluripotent stem cells by generating mitochondrial dysfunction and ER stress.^129^

#### 2.2.5 Anti‐apoptotic effects of HCMV

Despite the above‐mentioned association of HCMV with induction of apoptosis, HCMV mainly presents anti‐apoptotic effects. UL37 is one of the HCMV genes encoding for the anti‐apoptotic product, the so‐called localized apoptosis viral inhibitor in mitochondria (vMIA), because it prevents the release of mitochondrial CytC and therefore the activation of caspases with CARD type domains. Thus, vMIA ultimately increases the survival of infected cells.^130^, ^131^

Even though vMIA does not share homology with proteins of the Bcl‐2 family, its functions are similar since it prevents the permeabilization of the mitochondria, possibly by hijacking Bax oligomers, although it is also believed to have an effect on the function of Bak. The mechanisms by which vMIA exerts its anti‐apoptotic effects have not been fully elucidated; it may participate in inhibiting apoptosis through more than one pathway. vMIA is synthesized in the ER membrane, where it remains anchored through one hydrophobic N‐terminal end and transits through ER contacts with MAM. In the MAM, vMIA regions regulate calcium homeostasis and participate in cellular stress responses.^120^, ^132^

Another way HCMV fights cell stress and prevents apoptosis is by the use of the 2.7 kb RNA (β2.7), which is transcribed but does not code for functional translated products. The anti‐apoptotic effect of β2.7 RNA is given by its interaction with the mitochondrial CI, which is able to induce apoptosis under certain conditions like mitochondrial diseases and environmental factors. The β2.7 RNA specifically interacts with the subunit GRIM‐19 (genes associated with induced mortality by retinoids/interferon) essential for assembly and function of CI. Active CI supports the formation of the electrochemical gradient necessary for ATP production, so its interaction with the β2.7 RNA suggests that the virus stabilizes the gradient maintaining energy production. Moreover, it is well known that vMIA also promotes the stability of the mitochondrial membrane, being predominantly active in the later stages of infection; however, β2.7 RNA is abundantly expressed in much earlier stages of infection (12–24 h).^120^

Importantly, HCMV kills the neighbouring cells through a bystander effect, since HCMV has a wide range of anti‐apoptotic mediators that can prevent death in infected cells.^120^

Apparently, HCMV increases the expression of a wide variety of viral anti‐apoptotic mediators during the early stages of infection. The processes involved in viral entry into the host cell provide a transitory protection against cell death. Subsequently, certain genes are expressed to guarantee the latency of the viral genomes inside the cell. Indeed, if the virus is unable to inhibit these initial cell death induction events, the establishment of latency will be severely affected.^120^

#### 2.2.6 HCMV treatment in the paediatric population and mitochondrial involvement

Antiviral treatment is not recommended to prevent foetal infection during pregnancy, due to their teratogenic effects. Primary infected pregnant women are treated with acyclovir (not ganciclovir). The prophylactic efficacy of anti‐HCMV human gammaglobulin (Ig‐HCMV) has been evaluated in pregnant women with primary infection. Ig‐HCMV has been postulated to have immunomodulatory effects and reduce maternal viral load, decrease placental inflammation, and improve nutrition and foetal oxygenation.^126^, ^133^

All cases of congenital HCMV infection are not treated, only those with CNS involvement. Currently, the available antiviral treatment, ganciclovir/valganciclovir, is indicated in all children diagnosed during the first 6 months of life.^134^

Treatment with ganciclovir/valganciclovir in infants, even outside the neonatal period, has been shown to improve prognosis and minimize some of the sequelae (studies have demonstrated benefits from the treatment during the first month of life). However, treating these infected children involves an exposure to the potential toxicity of the drug for a longer period than in other indications (6 months).^135^, ^136^ On the other hand, despite the clinical manifestations of the potential toxicity of these drugs, the treatment is indicated. Ganciclovir/valganciclovir is guanosine analogues and, as such, has a therapeutic mechanism of action similar to that of NRTI ARV drugs. Ganciclovir/valganciclovir and NRTI act simultaneously as inhibitors and substrates of virus DNA polymerases, the enzymes responsible for synthesizing new nucleic acid chains, but also affect cellular DNA polymerases (both nuclear and mtDNA polymerases). mtDNA polymerase is the only enzyme responsible for the synthesis of the mitochondrial genome and it is more susceptible to being affected than nuclear polymerases, as its structure thrombocytopeniaand evolutionary origin are much closer to that of bacteria and viruses.

Despite the considerable data depicting NRTI‐derived mitochondrial dysfunction,^16^, ^17^ information regarding the potential mitochondrial toxicity of ganciclovir is scarce. Only one study reports secondary lactic acidosis related to ganciclovir in an adult patient that received this drug after kidney transplant.^137^ However, there are some studies showing that ganciclovir produces alterations in the mtDNA of senescent cells.^138^ The most common side effect of treatment with valganciclovir, already reported with ganciclovir, is neutropenia;^102^, ^126^, ^134^, ^139^, ^140^, ^141^ which occurs in up to two‐thirds of children treated for 6 weeks. Other less commonly reported side effects are, anaemia, nephrotoxicity, hepatotoxicity, fever and skin rashes.

The impact of the congenital infection has led to the need to develop consensus regarding the prevention of infection in pregnant women, the diagnosis during pregnancy and its treatment.^126^, ^139^, ^140^

Notably, the first months of life are a key stage in the child's development, in which the health problems will likely affect their future life.

### 2.3 Herpes simplex virus

Human herpes simplex virus (HSV) types 1 and 2 belong to the family *Herpesviridae*. HSV is a ubiquitous viral pathogen capable of causing both productive and latent infections in its human host.^142^ Infections are generally mild but can spread to the CNS, causing serious neurological damage. To enter its host, the virus must overcome a barrier of mucosal surfaces, skin or cornea. Keratinocytes are the main target during the initial entry to establish a primary infection in the epithelium, followed by a latent neuronal infection.^143^ They are especially contagious when symptomatic, but can also be transmitted in the absence of symptoms. Symptoms are usually painful vesicles or ulcers at the infected site, causing cold sores, genital herpes, keratitis, or encephalitis.^144^

#### 2.3.1 Structure and replication cycle

HSV presents a typical morphology with an icosahedral capsule of 162 capsomeres, covered with a viral envelope, and includes a genome comprising a single DNA molecule, from 120 to 250 kbp. The viral infection exhibits a definitive tendency toward tropism, being highly recurrent on the surfaces of organs that become infected. The productive phase of infection, where the virus releases multiple viral proteins, is followed by a latency phase in which the viral genome remains within host cells throughout the life of the infected individual. Occasionally, latent HSV can undergo reactivation processes and once again lead to a productive phase in which numerous viral proteins are released.^145^, ^146^

The HSV viral cycle can be divided into different phases including^146^ (i) entry phase; (ii) expression of viral genes, the glycoproteins and other virion components involved in the development of the infection, including UL41, which induces inhibition of host protein synthesis, destroying most of the mRNAs, allowing HSV to fully take over the protein synthesis machinery and increase efficiency of virus production and UL13 protein kinase, whose absence blocks infection. Once in the cell, the capsid passes through the nuclear pores and releases the DNA into the nucleoplasm. It is probable that the cellular cytoskeleton contributes to transport to the nucleus. (iii) Replication, carried out in the cell nucleus; (iv) combination, encapsidation and release, meaning that the linear viral DNA is packaged in a preformed capsid containing the viral protease; and (v) latency periods alternating with reactivation periods, in which the virus can be transported through the axon to the periphery, producing lytic infection at the level of the epithelial cells.

#### 2.3.2 HSV in the paediatric population

Neonatal HSV infection causes high mortality and significant morbidity. Incidence estimates range from 1/3000 to 1/20,000 births. Type 2 predominates over type 1 HSV.^147^ ^,^ ^148^ In the United States, paediatric HSV infections are common; as many as 36% of children <14 years of age have serologic evidence of HSV‐1 infection.^149^ Often around the age of 5, a child's first cold sore may appear. Cold sores (also called fever blisters or oral herpes) start as small blisters that form around the lips and mouth. After a few days, the blisters crust over and heal completely in a week.^150^

#### 2.3.3 Mitochondrial changes in HSV infection

HSV infection disrupts the oxidative balance within cells. Protein carbonylation, an irreversible modification that alters the conformation of proteins, and generally produces degradation by the proteasome, is an indicator of oxidative stress in cells. Specifically, HSV infection triggers an oxidative imbalance by depleting glutathione on entry.^151^ HSV causes oxidative stress and calcium release, as well as CytC release from mitochondria, thus aiding viral replication.^6^, ^151^ HSV suppresses cellular respiration by inhibiting electron transfer chain (ETC)^151^ and, more specifically, HSV US3 inhibits the transfer of electrons between CII and CIII.^151^, ^152^ On the other hand, HSV UL12.5 is a nuclease that is located in mitochondria, where it breaks down mtDNA. Finally, HSV UL7 traffics to mitochondria and interacts with adenine nucleotide translocator. The biological importance of this interaction and the UL12.5 catalysed loss of mtDNA for the growth of HSV is still unclear.^151^

Movement and mitochondrial distribution throughout the cytoplasm is crucial to maintain cellular homeostasis. Mitochondria are dynamic organelles but can be functionally altered during infection. HSV types 1 and 2 induce changes in mitochondrial morphology and distribution in the early and late stages of productive infection in human keratinocytes. A decrease in Δψm is observed within 2 h after infection and a decrease in cell vitality is observed 24 h after infection. Furthermore, the mitochondria migrate to the perinuclear area, where the HSV types 1 and 2 antigens are also observed, mainly in the early stages of infection. This indicates that HSV types 1 and 2 cause mitochondrial dysfunction in human keratinocytes.^143^

Of note, we were not able to identify any literature reporting HSV‐associated mitochondrial alterations in the paediatric population.

#### 2.3.4 HSV treatment in the paediatric population and mitochondrial involvement

Antiviral drugs, such as acyclovir, famciclovir and valacyclovir, are the most effective anti‐HSV drugs. However, although they can reduce the intensity and frequency of symptoms, they do not cure the infection^144^ (Table 5).

**TABLE 5:** Treatment of HSV in the paediatric population and mitochondrial involvement

Drug	Mechanism of action	Mitochondrial involvement	Paediatric population
Acyclovir	Inhibits viral DNA replication, interfering with viral DNA polymerase^153^, ^154^, ^155^	Degradation of mitochondrial DNA Inhibits mitochondrial ETC, between CII and CIII^151^	Common adverse effects in a cohort of infants treated with high‐dose acyclovir were: hypotension and seizures in 9% of infants; thrombocytopenia in 25% of infants; and elevated creatinine in 2% of infants, none of which developed kidney failure requiring dialysis. Many of the adverse effects reported in this cohort may be related to the underlying infection and not due to exposure to acyclovir^156^ Infants surviving neonatal HSV disease with CNS involvement had improved neurodevelopmental outcomes when they received suppressive therapy with oral acyclovir for 6 months^157^
Famciclovir (not approved in children)			iii. A single dose of oral famciclovir paediatric formulation was safe and well tolerated in infants 1–12 months of age with active, suspected, or latent HSV infection^158^
Valacyclovir (prodrug of acyclovir)			iv. Valacyclovir (15 mg/kg) was well tolerated in paediatric patients and demonstrated excellent bioavailability^159^

For non‐neurologic disease, antiviral agents such as acyclovir, famciclovir and valacyclovir are effective. For HSV encephalitis and neonatal disease, intravenous acyclovir is the drug of choice, although two other drugs, foscarnet and cidofovir, which have already been introduced in the previous HCMV section, can be used in the case of infection with acyclovir‐resistant viruses due to deficient thymidine kinase activity. Although intravenous acyclovir has dramatically improved the mortality associated with neuroinvasive HSV infection, the proportion of patients with subsequent permanent neurologic deficits remains high.^151^ Additional antiviral agents and combination therapies are needed to improve outcomes in these patients, especially, in the most vulnerable paediatric population.

#### 2.3.5 Paediatric studies of mitochondrial interaction in HSV infection

Neonates are particularly susceptible to severe HSV disease upon primary infection. Infants infected with HSV rarely develop disseminated, multi‐organ infections or encephalitis.^160^, ^161^ Life‐threatening HSV‐1 infections are also found in young children with inborn defects of innate immune signalling linked to type I IFN production. HSV infection causes leakage of mtDNA,^161^, ^162^ and it has been found that aberrant mtDNA packaging promotes escape of mtDNA into the cytosol, where it engages the DNA sensor cGAS (also known as MB21D1) and promotes STING (also known as TMEM173)‐IRF3‐dependent signalling to elevate IFN‐stimulated gene expression, potentiate type I IFN responses and confer broad viral resistance. In general, herpesviruses induce mtDNA stress, which enhances antiviral signalling and type I IFN responses during infection.^162^

### 2.4 Hepatitis viruses

Viral hepatitis is classified as acute (<6 months) and chronic (>6 months).^163^ Acute hepatitis involves a diffuse inflammatory process of the liver parenchyma secondary to the immune response to an offending agent. The hepatitis viruses (A, B, C, D and E) are mainly responsible for diffuse acute hepatitis, although they have suffered a drastic decrease in our environment.^164^ Only hepatitis B, C and D viruses can progress to chronic hepatitis and lead to the development of cirrhosis and hepatocarcinoma in the long term. All viral hepatitis produce similar symptoms that range from asymptomatic or non‐specific acute hepatitis, the most common presentation in previously healthy children, to fulminant forms, with acute liver failure.^164^ The transmission occurs enterally or parenterally depending on the virus: two agents are enterally transmitted: the hepatitis A virus (HAV) and the hepatitis E virus (HEV), while the remaining are mainly transmitted parenterally: hepatitis B (HBV), C (HCV) and D (HDV) viruses^165^ (Table 6). Despite their variability and taking into account that most primary infections are asymptomatic in children, during infection, there are different clinical stages including (i) initial or pre‐icteric period, which is nonspecific and of variable duration, with fever, asthenia, myalgias, headache and diverse digestive symptoms among others; (ii) icteric period, with appearance of jaundice; and (iii) posticteric or convalescent period during 2–4 weeks, with progressive disappearance of all symptoms.^164^

**TABLE 6:** Different viral hepatitis, their treatment and associated mitochondrial damage

Features	HAV	HBV	HCV	HDV	HEV
Transmission	Faecal—orally through contaminated water or food, favoured by overcrowdingand poor sanitary conditions^164^	Parenteral (vertical, horizontal by contact, by blood products or venous punctures and sexual)^164^			Faecal—orally through contaminated water or food, favoured by overcrowdingand poor sanitary conditions^164^
General characteristics	RNA virus *Picornaviridae* family Only a stable variant exists, very resistant to environmenttal conditions^164^	DNA virus *Hepadnaviridiae* family Up to eight different genotypes identified^164^	RNA virus *Flaviviridae* family Enormous genetic diversity and ability to constantly mutate, which favours persistence of infection Up to six genotypes and numerous subtypes HCV is not integrated into the genome of the host and the infection does not produce permanent immunity to reinfection by the same or another genotype^164^	Defective RNA, requiring the mandatory presence of HBV surface antigen for transmission in vivo^163^, ^164^ *Deltaviridae* family Circular RNA similar to plant viroids	RNA virus The major etiologic agent of non‐A enteric transmission hepatitis throughout the world^164^ Similar to *Caliciviridae* family
Epidemiology	The most frequent hepatitis in childhood, almost always benign and self‐limited Estimated 1.4 million yearly cases worldwide, at any age^164^	90% when acquired perinatally 20%–50% when acquired between 1 and 5 years 5% when acquired in adults 15% will present complications: cirrhosis or hepatocarcinoma HBV is still the main cause of cirrhosis worldwide^164^	HCV infection is estimated to affect 200 million people worldwide, and HCV antibodies are detected in 0.1%–0.4% of children in Spain^164^ It is usually chronic (50%), frequently following a course in the form of outbreaks or successive reactivations (this does not occur in children)^163^	4% of those chronically infected with HBV, become infected with HDV^164^	There are an estimated 20 million cases of HEV infection each year, of which 3.3 million develop symptoms^166^
Clinical data	The incubation period:15–50 days It is estimated that only 10%–30% of cases present with symptoms: jaundice with pale stools and dark urine, stomach ache and fever^163^, ^164^	If the immune response is ineffective, the infection will become chronic, and asymptomatic for many years^163^, ^164^ If the immune response is excessive, serious fulminant hepatitis and acute liver failure may occur The elevation of liver enzymes usually occurs between 2 and 6 months of age^164^	Most children with HCV infection are asymptomatic or have only mild nonspecific symptoms; progression to liver failure is exceptional Only 20% of children in the first 4 years of life present clinical symptoms or signs, hepatomegaly being the most frequent^164^	Acute hepatitis: Simultaneous infection with HBV and HDV can produce mild to severe, even fulminant, hepatitis, but recovery is usually complete and chronic hepatitis D is rare^167^	The infection is asymptomatic and self‐limited in almost all cases, except in immunosuppressed patients^164^ The incubation period after exposure to HEV ranges from 2 to 10 weeks. Jaundice and hepatomegaly are related symptoms^166^
Acute/chronic	Acute	Acute/chronic	Acute/chronic	Acute/chronic	Acute
**Prevention**	Multipurpose immunoglobulin^164^ Improvement of socio‐sanitary and hygienic conditions^163^ HAV vaccine	HBV vaccine^164^, ^168^	There is no specific vaccine or immunoglobulin^164^	Prevention and control of HDV infection are based on preventing transmission of HBV through vaccination^167^	Improvement of socio‐sanitary and hygienic conditions^166^
Treatment	There is no specific treatment for HAV. Symptoms may subside slowly, over several weeks or months. It is relevant to avoid unnecessary medications. Antiemetics and paracetamol should not be administered^169^	The treatment aims at the well‐being and nutritional balance of the patient. It is relevant to avoid unnecessary medications. Antiemetics and paracetamol should not be administered^168^ The FDA has licenced five drugs for the treatment of chronic HBV: ‐ IFN α for children> 12 years ‐ 3TC for children >3 years ‐ ADF for children >12 years ‐ Entecavir for children >16 years ‐ TDF for children >12 years^165^, ^168^	Administration of interferon during acute phase reduces the risk of chronification, with a cure rate of 90%,^164^ but direct‐acting antivirals are the best option nowadays, also in paediatrics Pegylated IFN α plus ribavirin^165^ The first direct‐acting antivirals used in the treatment of chronic HCV infection, telaprevir and boceprevir, were marketed in 2011. Their mechanism of action is to inhibit HCV protease and they were approved in combination treatment with pegylated interferon and ribavirin^170^	Current guidelines often recommend treatment with pegylated interferon alpha for a minimum of 48 weeks, regardless of the response observed during treatment^167^	There is no specific treatment that alters the evolution of acute HEV. As the disease usually remits spontaneously. Antiviral ribavirin may be useful in treating immunosuppressed patients with chronic HEV^166^
Mitochondrial damage	Oxidative stress by ROS generation^171^	Disrupts Δψm^172^ Proapoptosis^151^, ^173^, ^174^ Exerts substantial effects on mitochondria to change mitochondrial dynamics/signalling^175^, ^176^ Disrupts mitochondrial dynamics by inducing the translocation of dynamin‐related protein Drp‐1 to the mitochondria and subsequent mitochondrial fission^172^ Disrupts mitochondrial dynamics: induces fission and mitophagy to attenuate apoptosis^177^	ROS generation Inhibition of ETC CI^178^, ^179^ Increases Ca^2+^ from ER to mitochondria^151^, ^180^, ^181^, ^182^ Induces autophagy^182^	ROS generation Increased apoptotic ratios^183^	Inhibition of MRC CIII restricts HEV replication^184^
Paediatric population	Paediatric patients with acute HAV are at risk of increased oxidative stress, resulting in significantly lower levels of plasma antioxidants and increased lipid peroxidation. In the absence of other therapeutic options, antioxidant vitamin supplements could be given to help re‐establish the oxidant status balance^171^	The risk of developing chronic hepatitis varies from > 90% in newborns of mothers positive for HBV antigen (HBeAg), 25%–35% in children under 5 years of age and <5% in adults. HBeAg, a non‐particulate viral protein, is a marker of HBV replication. This is the only HBV antigen that crosses the placenta, leading to a lack of specific helper T‐cell response to the capsid protein and HBeAg in newborns. HBeAg is tolerated in the womb and acts as a tolerogen after birth. Perinatal transmission is frequent when mothers are HBeAg‐positive, whereas it occurs less frequently when mothers are HBeAg‐negative^185^	Mother‐to‐child transmission of HCV has become a leading cause of paediatric infection of HCV, and up to half of the children infected with HCV acquire the HCV infection in utero^186^	Most of the HDV‐IgG‐positive children show markedly elevated liver enzymes^187^	In many developing countries, anti‐HEV IgG seroprevalence studies show that most children under the age of 10 years have not been exposed to HEV. The seroprevalence increases dramatically between the ages of 15 and 30 years, and it plateaus at around 30%^188^

*Note*: Type F hepatitis is a very infrequent pathology, also triggered by viral infection, although only a few isolated cases have been documented in India, United Kingdom, Italy and France. Scarce data point to a mono‐stranded DNA and it has been classified as a type B hepatitis variant.

Abbreviations: 3TC, lamivudine; ADV, adefovir, CI, I complex; CIII, III complex; DNA, deoxyribonucleic acid; Drp‐1, dynamin‐1‐like protein; ER, endoplasmatic reticulum; ETC, electron transport chain; FDA, food and drug administration; HAV, hepatitis A virus; HBV, hepatitis B virus; HCV, hepatitis C virus; HDV, hepatitis D virus; HEV, hepatitis E virus; IgG, immunoglobulin G; INFα,interferon alpha; MRC, mitochondrial respiratory chain; RNA, ribosomal ribonucleic acid; ROS, reactive oxygen species; TDF, tenofovir; Δψm, mitochondrial membrane potential.

#### 2.4.1 Structure and replication cycle

Enteral hepatitis viruses replicate in the hepatocyte are excreted in the bile and are eliminated in the faeces. The virus binds to a receptor found on the surface of hepatocytes and other cells and spends its entire life in the cytoplasm, where it replicates using an RNA‐dependent polymerase encoded by itself. The young child behaves as a reservoir of the disease when the infection is asymptomatic, with viral elimination comparable to that of the common acute icteric presentation. For instance, HAV usually has a benign course and its evolution to chronicity has not been described. However, the fulminant form is the most serious complication with an estimated frequency of about 0.4% in children.^165^

Parenteral hepatitis viruses replicate in the liver after entering the body and, rather than through a cytopathic mechanism, histological damage depends on the activation of the host immune system, which causes destruction of liver cells. The clinical manifestations will depend on the intensity and duration of the abovementioned response.^164^ According to Center for Disease Control and Prevention data, 42% of chronic adult HBV infections have been acquired during childhood.^165^ However, thanks to universal vaccination against HBV, HCV is nowadays responsible for practically all chronic viral hepatitis in childhood in our setting. In childhood, the only route of HBV or HCV transmission in developed countries is vertical transmission during pregnancy or delivery from infected women. Breastfeeding does not seem to increase the risk of transmission to the child, despite the fact that HCV RNA has been detected in breast milk.^164^

#### 2.4.2 Hepatitis in the paediatric population

Despite the fact that HCV is responsible for practically all chronic viral hepatitis in childhood in the developed world, hepatitis A is the most frequent of all the viral hepatitis. The most relevant data regarding all types of infectious viral hepatitis and studies conducted in the paediatric population have been gathered in this review (Table 6).

#### 2.4.3 Mitochondrial changes in viral hepatitis

When considering the relationship between mitochondria and hepatic impairment in paediatrics, a bilateral association must be acknowledged, as primary mitochondrial disorders may produce a variety of hepatological problems in childhood^189^ and primary hepatitis is associated with mitochondrial dysfunction (Table 6).

HBV‐specific T cells present the ability to switch to OXPHOS in the absence of glucose and subsequently lead to increases in mitochondrial size and a lower Δψm, indicating mitochondrial dysfunction.^190^, ^191^

The most pronounced mitochondrial abnormalities observed in hepatocyte‐like degenerative cells in the course of chronic HBV infection are characterized by distinct inflammation, loss of mitochondrial ridges and the presence of myelin structures within the matrix.^192^, ^193^

On the other hand, HCV has been shown to induce mitophagy, although the precise underlying mechanism and the responsible effector protein remain unclear. The HCV 5A non‐structural protein plays a key role in the regulation of cellular mitophagy. Specifically, expression of HCV NS5A in hepatoma cells triggers distinctive features of mitophagy, including mitochondrial fragmentation, loss of mitochondrial membrane potential and Parkin translocation to mitochondria. Interestingly, NS5A expression concomitantly improves ROS production and treatment with an antioxidant attenuates the NS5A‐induced mitophagy event.^194^, ^195^ To better clarify this, a detailed summary of mitochondrial damage associated with each type of virus has been summarized (Table 6).

The mitochondrial interactions of the remaining hepatitis virus have not been reported in the literature so far, but they should not be ruled out and further studies are needed in that direction.

#### 2.4.4 Hepatitis treatment in the paediatric population and mitochondrial involvement

In general, no etiological treatment is considered against chronic HBV and HCV hepatitis in paediatrics. The child can return to normal activity over the course of several weeks. Hospitalization usually is not necessary, except for surveillance of young infants or when progression to fulminant hepatitis is suspected.^163^, ^164^

In acute non‐remitting HCV treatment with interferon may be indicated,^164^ which has been associated with mitochondria due to its antioxidant capacity.

In HBV, the option of choice consists of two NRTIs: adefovir (ADV) and TDF, both of which cause mitochondrial dysfunction in renal tubular cells and reprogramming of glucose metabolism.^196^ TDF has previously been discussed in the HIV section (Table 2) due to its potential off‐target inhibition of human gamma DNA polymerase, involved in the replication of mtDNA. A depletion of intracellular mtDNA levels can lead to variable clinical manifestations of mitochondrial toxicity (neuropathy, myopathy, lactic acidosis), but these side effects have been very rarely reported with oral antiviral agents active against HBV. ADV and TDF are associated with dose‐dependent, but generally reversible, proximal renal tubular toxicity.^196^ For these reasons, patients receiving these agents should be monitored for renal toxicity and the dose modified for renal failure.

20%–25% of HIV‐positive patients are co‐infected with HBV or HCV, and these patients have increased sensitivity to liver toxicity from ARV as compared to mono‐infected patients. The relationship between high ARV concentrations and toxicity has been clearly demonstrated with certain PIs and NNRTIs that have a predominantly hepatic metabolism and which have also been related to mitochondrial apoptosis. NRTIs are not predominantly metabolized by the liver, but may be toxic to the liver through mitochondrial involvement. In any case, rigorous monitoring is essential^197^ taking into account the specific mitochondrial interactions of such treatment options (Table 6).

#### 2.4.5 Paediatric studies of mitochondrial interaction in hepatitis infection

Some hepatitis viruses, such as HAV, induce oxidative stress in children. The main sources of ROS in hepatocytes in acute or chronic disease are mitochondria and cytochrome P450 enzymes. Oxidative stress, as the consequence of increased intracellular ROS concentrations, can be reduced by antioxidants, such as vitamin A, vitamin C, vitamin E and reduced glutathione.^171^, ^198^

## 3 MATERIALS AND METHODS

We searched for scientific publications in three main database sources including Pubmed (MEDLINE), Web of Science and SCOPUS. We included the common search terms: ‘mitochondria AND paediatric OR childhood OR infant OR children’ for all the infectious diseases. For each infectious disease we added the following terms: AIDS‐HIV, human immunodeficiency virus; human cytomegalovirus (HCMV); herpes, herpes simplex virus (HSV); hepatitis, hepatitis A virus (HAV), hepatitis B virus (HBV), hepatitis C virus (HCV), hepatitis D virus (HDV), hepatitis E virus (HEV); adenovirus; T‐cell lymphotropic virus‐1 (HTLV‐1) and influenza virus. We reviewed publications in English between 1984 and 2020. We used the Rayyan QCRI software for systematic reviews (http://rayyan.qcri.org), a free web and mobile app, that helps expedite the initial screening of abstracts and titles using a process of semi‐automation while incorporating a high level of usability.^199^ The studies were assessed for relevance and were blind selected by two independent investigators (SR and CM). With respect to the inclusion criteria all randomized controlled studies in human models were included, as well as, case reports and review articles. Animal models were excluded for this review.

## 4 RESULTS AND DISCUSSION

A summary of the viruses, related mitochondrial interactions and paediatric studies available is provided in (Table 7).

**TABLE 7:** Summary of different viruses, their therapies and mitochondrial targets including studies in the paediatric population

Virus	Medical need for new therapy	Current antiviraltherapies	Limitations of current therapies	Potential target	Known target		Metabolic or mitochondrial function
Adenovirus	Solid organ transplant patients Pulmonary, gastrointestinal Disseminated disease	Cidofovir Lipophilic Cidofovir	Bone marrow suppression Nephrotoxicity	E1B‐19K	Bax Bak Bik BNip p53		Anti‐apoptosis
Paediatric studies	Adenovirus infections are more common in young children, due to lack of humoral immunity; more than 80% of diagnosed adenovirus infections occur in children <4 years, most of whom do not require treatment. Cidofovir is the drug of choice for severe infections,^200^ although it is significantly associated with nephrotoxicity^151^ E1B‐19K, one of the adenoviral oncogenes, counteracts E1A‐induced apoptosis during adenovirus infection. E1B‐19 is located in the mitochondria during the early and late stages of adenovirus infection. E1B‐19K was the first Bcl‐2 viral homologue to be discovered. It possesses BH1, BH2 and BH3 domains and inhibits apoptosis induced by p53 activation triggered by E1A adenovirus, stimulation of TNFα and Fas, induction of TGF‐β, ultraviolet radiation and DNA damaging agents. E1B‐19K can interact with p53 and suppress p53‐induced mitochondrial mediated apoptosis. By dual interaction with p53 and Bak, E1B‐19K can prevent Bak activation as well as Bak dependent activation^151^, ^201^, ^202^, ^203^						
Enteroviruses (Nonpolio)	Neonatal sepsis Myocarditis Aseptic meningitis Meningoencephalitis Upper respiratory infections	None Pleconaril (out of market)	N/A	Non‐structural protein 2B	Viroporin		Antiapoptosis increased ER Ca^2+^ efflux, Decreased mitochondrial Ca^2+^ uptake
Paediatric studies	Neonates and young children are at the greatest risk of developing severe and occasionally fatal enteroviral infections.^151^, ^204^ Pleconaril, which was molecularly engineered to block enterovirus binding to host cells, has been studied in the setting of upper respiratory disease, aseptic meningitis and neonatal sepsis^151^ Enterovirus 71 2B protein localizes in the mitochondria and induces cell apoptosis by interacting directly with and activating the pro‐apoptotic protein Bax. 2B recruited Bax to the mitochondria and induced Bax conformational activation. In addition, mitochondria isolated from 2B‐expressing cells that were treated with a recombinant Bax showed increased Bax interaction and CytC release in children^205^						
HBV	Chronic hepatitis	INF α Pegylated IFN‐α 3TC, ADF, TDF ADV Entecavir Telbivudine Emtricitabine	Variable clinical response Antiviral Resistance Toxicities: Flu‐like symptoms Nephrotoxicity Musculoskeletal	HBx	VDAC3		Disrupts ΔΨ_m_ Proapoptosis
Paediatric studies	i. In HBV‐infected children, the level of oxidative stress markers correlates with the rate of chronicity of the disease. The direct mechanisms underlying this effect are not known^206^, ^207^ ii. IFN‐α‐2b, pegylated IFN‐α‐2a and 3TC are FDA approved for treatment of children and adults, although response rates are poor (approximately 25%–30% overall). Multiple nucleoside analogues (3TC, entecavir and telbivudine) and a nucleotide analogue (ADV) are FDA‐approved for treatment of adults, and emtricitabine has also been utilized (although not FDA‐approved), but safety and efficacy in children have not been established^151^						
HCV	Chronic hepatitis	INF α, DAA Pegylated IFN‐α Ribavarin	Variable clinical response Toxicities: Flu‐like symptoms Haematologic Neuropsychiatric	Core protein	MOM permeabilization opening		ROS generation Inhibition of ETC CI Increase Ca^2+^ from ER to mitochondria
Paediatric studies	Neutrophil involvement occurs in the pathogenesis of chronic HCV in children. Neutrophils undergo increased expression of TLR2 and TLR4 (which correlates with the characteristics of hepatocytic damage and necrosis enhancement), inhibition of oxygen metabolism, and, after TNF‐alpha preactivation, increased ROS production^208^						
HSV	Neonatal CNS Disseminated disease Meningoencephalitis Genital disease Keratitis	CNS: Acyclovir Foscarnet Cidofovir Non CNS: Acyclovir Famciclovir Valacyclovir Ophthalmic: Trifluridine Idoxuridine Vidarabine	CNS disease: High morbidity Toxicities: Bone marrow suppression Haematologic Nephrotoxicity Electrolyte imbalance	UL7 UL12.5 US3	ANT2 MtDNA ETC		Degradation of mtDNA Inhibits ETC, between CII and CIII
Paediatric studies	The virus is generally acquired during childhood and produces lifelong infections due to its ability to infect and remain dormant in neurons. There is accumulated evidence that suggests that HSV‐1 infection in the brain, in both symptomatic and asymptomatic children, could lead to neuronal damage and ultimately neurodegenerative disorders. Possible cellular and molecular mechanisms that lead to neurodegeneration are, for example, protein aggregation, autophagy dysregulation, oxidative cellular damage and apoptosis, among others^209^						
HCMV	Congenital infection Pulmonary Gastrointestinal Hepatic, retinal and disseminated disease in immunocompromised hosts	Ganciclovir Valganciclovir Cidofovir Foscarnet Maribavir Ophthalmic: Valganciclovir Formivirsen	Antiviral resistance Toxicities: Bonemarrowsuppression Haematologic Nephrotoxicity Electrolyte imbalance	pUL37x1/vMIA β2.7 RNA Warburg effect TCA cycle	Bax GRIM‐19 complex		Anti‐apoptosis ER Ca^2+^ efflux Regulates mitochon‐drial HtraA2/Omi Inhibits ATP synthase
Paediatric studies	Congenital HCMV infection can cause serious brain abnormalities. Apoptotic brain cells infected with HCMV have been detected in infants with congenital infection. Surprisingly, its well‐known anti‐apoptotic genes, including pUL37x1 or vMIA, protect infected human fibroblasts from apoptosis and caspase‐independent mitochondrial serine protease. Although pUL37x1/vMIA was shown to be protective in fibroblasts, it does not protect human neural precursor cells infected with HCMV from cell death under physiologically relevant oxygen stresses^210^						
HHV‐8	Kaposi sarcoma Lymphoproliferative disease in HIV co‐infected patients	None	N/A	Warburg effect K7 K15 KSBcl2	Bcl‐2, active caspase‐3 HAX1		Required for latency
Paediatric studies	Endemic Kaposi's sarcoma is a common disease of children in sub‐Saharan Africa and was documented before the introduction of HIV. Like other herpes viruses, HHV‐8 has the ability to escape the host's immune response during initial infection, during sustained latency and during reactivation. The host uses two levels of defence to counter microbial infection; the innate immune system and the adaptive immune system HHV‐8 has developed multiple molecular mechanisms to evade host immunity. MAVS has been observed to participate^211^						
HIV	AIDS	NRTI NNRTI PI II	Failure to eradicate infection Antiviral resistance Adherence Toxicities: Gastrointestinal Haematologic Metabolic Cardiovascular	Vpr	VDAC ANT3	i. Promotes PTP opening ii. ΔΨ_m_ loss	
Paediatric studies	MtDNA levels are lower in HIV‐positive patients exposed to HIV than in HIV‐uninfected children. Peripheral blood mononuclear cell mtDNA levels are significantly altered in infants exposed to ARVs, not infected with HIV, and their infected mothers compared to infants and women not exposed to ARVs. At 5 years, peripheral blood mononuclear cell mtDNA levels increase to normal concentrations in children exposed to ARV but remain depressed in children not exposed to ARVs^212^						
HTLV‐1	ATLL Spastic paraparesis	INF‐α Nucleoside analogues		p13		i. Rapid mitochondrial K^+^ influx ii. Depolarization iii. Alteration of mitochondrial Ca^2+^ uptake	
Paediatric studies	The regulatory non‐structural proteins of HTLV‐1, p13II, are associated with MIM, where it is proposed to function as a potassium channel. The entry of potassium through p13II into the matrix causes depolarization of the membrane and triggers processes that lead to T‐cell activation or cell death through apoptosis.^213^, ^214^						
Influenza	Upper and lower respiratory tract infections Sepsis‐like syndrome	Amantadine Rimantidine Oseltamivir Zanamivir Peramivir	Antiviral resistance Need for IV formulations for severe disease	PB1‐F2	VDAC1 ANT3 Non selective ion channel	ΔΨm dissipation PTP opening Pro‐apoptotic	
Paediatric studies	The virus can also reach the lower respiratory tract (trachea, bronchi and lung alveoli) in infections with pandemic strains, especially in children and the elderly. HHV‐8 shows the participation of MAVS.^215^						

Abbreviations: 3TC, lamivudine; ADV, adefovir; ANT, adenine nucleotide translocator; ARV, antiretrovirals; ATLL, Adult T‐cell Leukemia/Lymphoma; CI, complex I; CII, complex II; CIII, complex III; CytC, cytocrom C; CNS, central nervous system; ER, endoplasmàtic reticulum; ETC, electron transport chain; FDA, Food and Drug Administration; HAM, HTLV‐Associated Myelopathy; HBV, hepatitis B virus; INF α, interferon alpha; HCMV, human cytomegalovirus; HCV, hepatitis C virus; VDAC, voltage‐dependent anion channel; HHV‐8, human herpesvirus type 8; HIV, human immunodeficiency virus; HSV, herpes simplex virus; HTLV‐1, human T‐cell lymphotrophic virus; IMM, inner mitochondrial membrane; II, integrase inhibitor; IV, intravenous; KSHV, Kaposi sarcoma‐associated herpesvirus; MAVS, mitochondrial antiviral signalling protein; mtDNA, mitochondrial DNA; NNRTI, Non‐nucleoside Reverse Transcriptase Inhibitor; NRTI, Nucleoside Reverse Transcriptase Inhibitor; OMM, outer mitochondrial membrane; PI, protease inhibitor; PTP, permeability transition pore; pUL37x1, UL37 exon 1 protein; ROS, reactive oxygen species; TCA, tricarboxylic acid; TNF, tumour necrosis factor; TLR2, toll‐like receptor receptor 2; VMIA, viral mitochondria‐localized inhibitor of apoptosis; ΔΨm, mitochondrial membrane potential. Adapted from Williamson et al.^151^

The outcomes displayed have been obtained using Rayyan QCRI software. The item *HIV* showed 229 unique entries in Rayyan, 35.8% of which were included among the latter, 20.7% were comparative studies, 2.4% corresponded to trials, 1.2% were controlled studies and 2.4% corresponded to randomized studies. The item *HCMV* showed 32 entries, 25% of which were included. The item *Herpes* showed 17 unique entries in Rayyan (5.9% included). The item *HVC* showed 145 unique entries in Rayyan (10.3% included). There is wide evidence and a large number of studies reporting information about mitochondrial dysfunction associated with HIV and HCMV infections, while data on other infections are scarce.

The question of the contribution of cellular metabolism to viral propagation and their association with mitochondria was raised more than 60 years ago, but up to the present date, no review on the association of mitochondrial dysfunction and the most relevant viral infectious diseases in the paediatric population has been conducted. Importantly, mitochondrial interactions and toxicity are ultimately determined by both viral load and the antiviral drugs used, and often turn out to be reversible once the toxic agent is interrupted.

As obligate intracellular parasites, all viruses rely on their host's metabolic functions, and hence they manipulate these functions to varying degrees. Some viruses have developed mechanisms to ensure cellular survival through an ongoing energy supply during the entire replication cycle and/or countermeasures to the antiviral activity exerted by mitochondria. The latter is reflected by the clustering of mitochondria around replication sites of several viruses. This finding supports the so far disputable notion of a direct transfer of ATP from mitochondria to virus factories. Most viral infections simply consume cellular resources and are therefore associated with the induction of oxidative stress. In fact, we have reviewed the increase in mitochondrial ROS observed during the course of several viral infections, such as HIV, enteroviruses, HSV, meningitis and hepatitis. Interestingly, ROS is not only a by‐product of oxidative respiration but also regulates signalling pathways such as signal transducer and activator of transcription and phosphoinositide 3‐kinase pathways. Hence, an increase in ROS as a cellular stress signal has to be avoided or counteracted by slow virus replication or the establishment of a persistent infection.^216^ Moreover, many infectious processes caused by different pathological agents are not only related to ROS overproduction but also share some other same molecular events, such as cell death and inflammatory mechanisms. For instance, in herpes, HCMV, or HIV infections, cells die by caspase‐mediated apoptosis induced by cytotoxic T cells. In particular, in both HSV and HCMV infections, there is an up‐regulation of caspase‐3 and not of caspase‐6 from mitochondria in the infected cells.^217^

Mitochondrial features change with age, and therefore to review molecular events in the young patients is important and could determine the future of the patients. When referring to specific alterations of the MRC, given infections (e.g., HIV) in children have been associated with decreased levels of different complexes of the mitochondrial ETC. There are controversial data reporting such impairment as an independent event from the observed depletion of mtDNA levels.^104^, ^216^ As observed in this review, not only the pathogens but also their treatments are frequently associated with mitochondrial changes. In this line, alterations in healthy infants exposed to ARVs have been reported.^40^ Moreover, the reduction in activity of CI, CIII, and CIV and in general mitochondrial oxygen consumption rates in HIV infected paediatric patients either on or off treatment (in comparison to the healthy control population) is not attributable to a dysfunction of a single respiratory chain complex or a reduction of their protein synthesis rate.^216^

In clinical practice, it is often difficult to differentiate whether mitochondrial abnormalities are exclusively related to the infection itself (e.g., HIV) or its treatment (e.g., NRTI).^2^ Importantly, these abnormalities have been correlated with the onset of clinical symptoms in the paediatric population; that is, mitochondrial alterations are more evident in children presenting clinical manifestations (such as lipodystrophy under HAART) than in those who do not.^104^ Co‐infection with HIV/HCV is a main issue also in the paediatric population^218^ and primary hepatitis is related to mitochondrial dysfunction, specifically OXPHOS and Δψm alterations.^189^, ^190^, ^191^ In many viral infections, mitochondrial abnormalities can also lead to long‐term metabolic complications,^110^ emphasizing the importance of longitudinal studies assessing mitochondrial changes and derived clinical consequences over time in the paediatric population. NRTIs still represent the option of choice as the core treatment in several viral infections in children, including HIV, HCMV and HCV, among others. When acting simultaneously as inhibitors and substrates of the virus polymerases, they may lead to interference in mitochondrial genome replication as well. To mention just a few examples, such is the case with 3TC, TDF, or ZDV use against HIV,^2^, ^10^, ^61^ ganciclovir, valganciclovir, cidofovir use against HCMV,^135^, ^136^ or telaprevir and boceprevir use against HCV.^170^ Another example of oxidative balance disruption and inhibition of mitochondrial ETC is HSV infection. Specifically, HSV US3 protein inhibits electron transfer between CII and CIII.^151^, ^152^ Moreover, HSV types 1 and 2 induce changes in mitochondrial morphology and distribution in the early and late stages of productive infection in human keratinocytes,^143^ evidencing the fact that mitochondrial affectations are not limited to molecular disruption but also to ultrastructural changes.

Sometimes the mitochondrial and cell changes triggered by the infective process are aimed at protecting the cell.

Importantly, in other cases, mitochondria of the infected cell turn out to be the main therapeutic target to treat the infection and pharmacological inhibition of a given mitochondrial performance may represent a key step to avoid pathogen replication. Such is the case of pharmacological inhibition of complex III in HEV. Mitochondria‐targeted pathogen products and the mitochondrial pathways affected by them provide potential novel targets for the rational design of drugs. Pathogen products may alter oxidative balance, mitochondrial PTP, ΔΨm, ETC and ATP production.^151^ The finding that blocking of these functions inhibits pathogen growth in many systems suggests that drugs designed to affect viral mitochondrial products, or their targets will be effective in inhibiting the targeted pathogen. Understanding the mechanisms underlying the effects of viral mitochondrial products and their targeted pathways will enable rapid and efficacious drug design.

**FIGURE 5:**
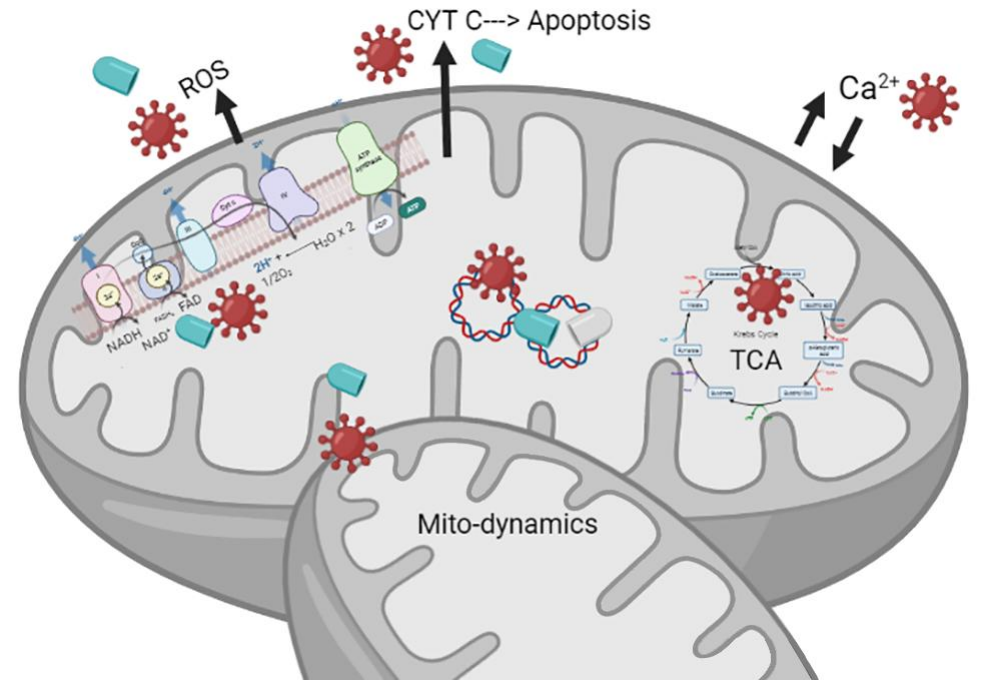
General summary of the main mitochondrial changes associated to viral agents and antiviral drugs, described in the present review. To summarize, all viruses herein depicted are related to apoptosis and subsequent ROS production, often related to mitochondrial respiratory chain dysfunction. Specifically, HIV is able to promote metabolic changes and HIV‐infected (and bystander) cells undergo apoptosis, present imbalance between oxidants and antioxidants, and Ca^2+^ overload, as an HIV‐derived toxic effect. HCMV, which presents both anti‐ and pro‐apoptotic properties, also affects cell metabolism, and induces mitochondrial biogenesis and respiration, to facilitate its own replication, which otherwise triggers increased ROS. HSV is associated with inhibition of mitochondrial respiratory chain between CII and CIII, ROS/Ca^2+^ overload and CytC release. HV affects mito‐dynamics by promoting mitochondrial fragmentation and changes in mitochondrial morphology and mitophagy, in association with ROS generation. On the other hand, anti‐HIV/anti‐HCMV/anti‐HV NRTIs are classically associated to mtDNA depletion, due to off‐target inhibition of endogenous polymerases, whereas protease inhibitors are associated with mitochondrial network fragmentation (mito‐dynamics), apoptosis and ROS/calcium generation. Ca^2+^, calcium; CytC, cytochrome C; Mito‐dynamics: mitochondrial fusion, mitochondrial fission and mitochondrial transport; ROS, reactive oxygen species; TCA, tricarboxylic acid

Mitochondrial performance is highly adaptive during a viral infectious process. For instance, during hepatitis viruses infections, switching to OXPHOS in the absence of glucose and the subsequent increase in mitochondrial size and a lower Δψm has been documented.^190^, ^191^ Figure 5 provides a summary of all mitochondrial changes derived from both viral and antiviral agents (Figure 5). In line with these metabolic arrangements, immunometabolic mechanisms should also be taken into account. Although there is a lack of data in the children, interesting studies have found alterations in mitochondrial biogenesis in neurons and astroglia, which could ultimately modulate neuroinflammation processes together with immunometabolic imbalance in the brain derived from ARV have been reported.^219^ Several studies indicate that upon activating glial cells, HIV proteins induce metabolic and inflammatory responses.^220^ The metabolic responses include alterations in ROS, ATP production, lactate production, oxygen consumption and autophagic flux. These metabolic changes precede, or are concomitant with, induction of inflammatory gene expression.^221^

In most cases, the number of children infected and receiving drug therapy against a given infection is increasing. Also, it is likely that if treatment ends up as indicated in pregnant women with acute infection, the number of treatment‐exposed newborns will also increase. Since studies and information are limited, especially in children; it is essential to accurately assess the potential mitochondrial toxicity of such pharmacologic therapeutic agents in a population as susceptible as newborns and infants.

In the near future, the identification of pathways or metabolites that are common to multiple viruses and pathogens remains an important challenge. Additionally, metabolic alterations that are directly involved in pathogen replication and not just a consequence of the infection need to be identified. There are more data to come on the complex interaction between pathogens with mitochondrial metabolism.^216^ It is known that disruption of mitochondrial integrity has been identified as a key virulence strategy of several viral pathogens.^222^

## 5 CONCLUSION

As a summary of the topics herein discussed:

‐ Infants are a susceptible population group, which is especially vulnerable during specific infective processes.
‐ Despite the great number of studies in HIV and HCMV, there is a lack of mitochondrial studies in most infective processes in the paediatric population.
‐ Mitochondria are a main player in specific infections, due to (i) the reported molecular and ultrastructural alterations directly derived from the viral pathogen, (ii) the reported molecular and ultrastructural changes derived from the antiviral treatment, (iii) their role as a therapeutic target in the disease, (iv) their implication and correlation in further clinical manifestations, (v) their identification as a key virulence strategy of infective pathogens, (vi) their high adaptability during the infection process and (vii) their protective role during the infectious process.
‐ Once a given mitochondrial toxic agent (either the pathogen or its treatment) is withdrawn, the observed lesions are likely to be restored.
‐ There is an urgent need to carry out longitudinal studies monitoring long‐term effects in the grown‐up children.

## AUTHOR CONTRIBUTIONS

Constanza Morén and Sonia Romero‐Cordero conceived the manuscript. Clàudia Fortuny and Antoni Noguera‐Julian contributed with paediatric and clinical knowledge. Francesc Cardellach contributed with mitochondrial and clinical knowledge. Francesc Cardellach and Constanza Morén supervised the development. Sonia Romero‐Cordero and Constanza Morén searched databases and performed double blinding selection. All co‐authors contributed to the supervision of the information gathering and writing process.
